# Advances in the Stability of Halide Perovskite Nanocrystals

**DOI:** 10.3390/ma12223733

**Published:** 2019-11-12

**Authors:** Maning Liu, Anastasia Matuhina, Haichang Zhang, Paola Vivo

**Affiliations:** 1 Faculty of Engineering and Natural Sciences, Tampere University, P.O. Box 541, FI-33014 Tampere, Finland; maning.liu@tuni.fi (M.L.); nastia.matuhina@gmail.com (A.M.); 2 Key Laboratory of Rubber-Plastic of Ministry of Education (QUST), School of Polymer Science and Engineering, Qingdao University of Science and Technology, Qingdao 266042, China; haichangzhang@hotmail.com

**Keywords:** perovskite nanocrystals, stability, lead-free, encapsulation, ligands, degradation, all-inorganic perovskites, lower dimensional perovskites

## Abstract

Colloidal halide perovskite nanocrystals are promising candidates for next-generation optoelectronics because of their facile synthesis and their outstanding and size-tunable properties. However, these materials suffer from rapid degradation, similarly to their bulk perovskite counterparts. Here, we survey the most recent strategies to boost perovskite nanocrystals stability, with a special focus on the intrinsic chemical- and compositional-factors at synthetic and post-synthetic stage. Finally, we review the most promising approaches to address the environmental extrinsic stability of perovskite nanocrystals (PNCs). Our final goal is to outline the most promising research directions to enhance PNCs’ lifetime, bringing them a step closer to their commercialization.

## 1. Introduction

In the last decade, metal halide perovskites semiconductors have received tremendous attention worldwide because of their outstanding optoelectronic properties, namely the high absorption coefficients, long charge-carrier diffusion lengths, exceptional high defect tolerance, tunable optical bandgaps, and moderately high charge-carrier mobility [1,2]. Such versatile characteristics of perovskites, together with their low formation energies, their suitability with high-volume manufacturing techniques, and their being made of low-cost and earth-abundant elements, make perovskites superstar materials for optoelectronic devices and particularly for photovoltaics, for which the certified power conversion efficiencies (PCEs) up to 25.2% [3] have been recently achieved at a skyrocketing pace. On the other hand, the success of conventional chalcogenide nanocrystals and semiconductor quantum dots (QDs) (e.g., CdSe, CdS, ZnS) [4] has raised interest to develop perovskites at nanoscale. Their facile synthesis along with their exceptional optoelectronic properties, have significantly boosted their use for several applications, including light-emitting diodes (LEDs), photodetectors, and lasers [5]. The quantum confinement allows the tunability of the optoelectronic properties of perovskite nanocrystals (PNCs) according to the size, defined in terms of their Bohr radius [6]. This opens up an array of properties and possible applications. PNCs, with at least one dimension below 100 nm per unit crystal cell [6], have widely achieved enormous interests with more than 800 papers published in 2018 [7]. However, similarly as their parent perovskite materials, they also suffer from instability inherent to the ionic lead (Pb)-halide bond and the dynamic nature of the ligand binding [6]. Other bottlenecks of PNCs impeding their commercialization mainly include the toxicity of Pb and the challenge to integrate them into macroscopic devices [8,9].

PNCs’ stability is mainly influenced by both composition (cations and halide anions) and atmospheric factors. The strong ionic bonding of halide PNCs inherently results in their easy reactivity with moisture, oxygen, temperature, and UV light, requiring severe conditions for their storage and further applications [10,11]. Over the past few years, many strategies have been developed and employed to improve PNCs stability during and/or after their synthesis. For instance, the synthesis of PNCs with low dimensionality of the inorganic part [8,9], surface treatment with hydrophobic and strong electron-withdrawing molecules [10], and capping perovskites with polymers or inorganic oxides [11,12], have been effectively used to enhance the environmental stability of as-formed colloidal PNCs and their corresponding optoelectronic devices.

In this review, we focus on the emerging new strategies to improve PNCs stability in such a dynamic and fast-moving field. While many good reviews have been published on PNCs so far [5,6,12,13], a comprehensive overview with specific emphasis on their stability issues and the most recent approaches to address them is not yet thoroughly reported. We aim at filling this knowledge gap by surveying various strategies to boost PNCs stability, with a major highlight on the intrinsic chemical- and compositional- factors. In Section 2, we focus on the most recent trends in the colloidal synthesis and post-synthesis treatments to increase PNCs stability. Emphasis is put on the selection of organic ligands that greatly influence the size and the shape of PNCs and passivate the surface, thus strongly affecting PNCs stability. In Section 3, we examine each constituent of PNCs structure and its role in the overall stability. We also provide a brief overview on the most promising Pb-free PNCs in terms of optoelectronic properties and PNCs inherent stability. In Section 4, we report about the recent advances on the approaches to address the environmental stability of PNCs, i.e., the effect of heat, moisture, oxygen, and UV light on PNCs degradation. Finally, we provide our views on future goals to advance the state-of-the-art on PNCs extrinsic stability. We believe that this work will be a useful reference for researchers working in the field, eventually promoting new insights and future studies to significantly enhance the stability of PNCs, thus bringing them closer to the commercialization.

## 2. Synthesis and Post-Synthesis Treatments

In this section, we will briefly discuss the advances towards stability improvement upon the significant synthetic routes that have been developed within injection methods. Moreover, we provide an overview of several key strategies for post-synthesis treatments to improve the stability of as-synthesized colloidal PNCs, mainly including surface passivation, phase transition, and ions exchange.

### 2.1. Key Synthesis Methods

The widely used colloidal synthesis approaches in solution process provide an effective route for the manufacturing of uniform and high-quality PNCs. To date, the most employed synthetic routes for PNCs are the injection methods, which can be divided into hot injection route and low temperature injection process [14,15]. Upon different synthesis approaches, by controlling reaction temperature, capping ligands and other reaction conditions, various halide PNCs can be achieved with variable components (e.g., organic/inorganic hybrid or fully inorganic), morphological dimensionalities from zero to three dimension (0D–3D), sizes and optical properties, which are closely in relation to the stability issue of the as-prepared colloidal PNCs.

#### 2.1.1. Hot-Injection Method

The high temperature hot-injection procedure has been commonly employed for the synthesis of conventional metal chalcogenide (e.g., CdSe, CdS, PbS) nanocrystals [16], and is also widely used to synthesize metal halide PNCs, particularly for fully inorganic ones due to the high temperature reaction. Kovalenko et al., firstly fabricated lead-based CsPbX_3_ (X = Cl, Br, I and the mixture) PNCs with a typical hot-injection method [17]. In general, the formation of CsPbX_3_ NCs is initiated by the swift injection of Cs-oleate into a precursor (e.g., an octadecene solution) containing PbX_2_ and long-chain alkyl acid and amine, i.e., oleic acid (OA) and oleylamine (OAm), at high temperatures normally in the range of 140–200 °C. The function of the mixture of OA and OAm is to solubilize PbX_2_ in precursor and accordingly to stabilize the as-formed PNCs [14]. Instead of the very common source of cesium oleate, i.e., cesium carbonate in OA, some other Cs-containing compounds such as cesium stearate and cesium acetate can produce improved processability and smaller sizes for PNCs due to the higher solubility of acetate or stearate based precursor [18,19]. The overall formation process of CsPbX_3_ can be expressed by Equation (1):

(1) $$2\text{Cs-oleate}\;+\;3{\mathrm{PbX}}_{2}\;\to \;2\mathrm{Cs}{\mathrm{PbX}}_{3}\;+\;\mathrm{Pb}{\left(\mathrm{oleate}\right)}_{2}$$

**Figure 1:**
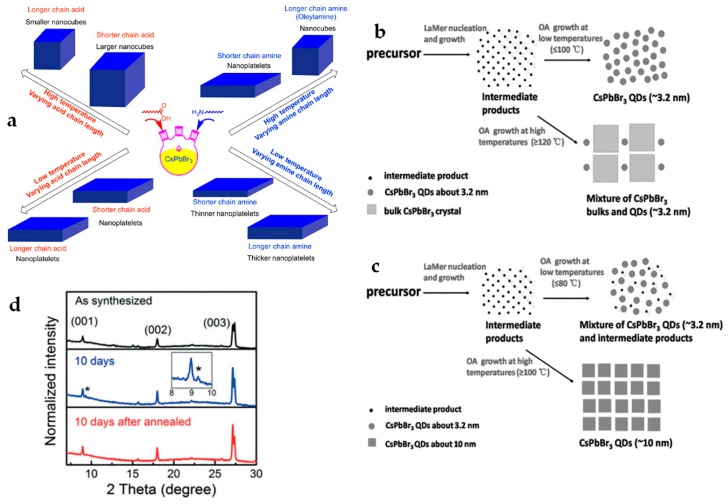
(**a**) Summary of the shape and size dependence on the chain length of carboxylic acids and amines. Reproduced with permission [19]. The growth processes of CsPbBr_3_ quantum dots (QDs) in (**b**) tetra-ethylene glycol dibutyl ether (EGDBE) with the high polarity, and (**c**) EGDBE with the low polarity at different temperatures. Reproduced with permission [22]. (**d**) X-ray diffraction (XRD) patterns of as-synthesized Cs_3_Bi_2_X_9_ NCs (black), Cs_3_Bi_2_X_9_ NCs stored in open air for 15 days before (blue) and after annealing (red). There is no other observed peak at 2θ higher than 30° in the complete pattern. Reproduced with permission [23].

Basically, PbX_2_ is the only source of X^−^ ions and thus one third of Pb^2+^ in precursor is consumed for the byproduct of lead oleate. In this case, lead-to-cesium molar ratio (R_Pb-Cs_) is one key factor to influence the formation of desired CsPbX_3_ NCs, which generally requires the value of R_Pb-Cs_ to be 1.5 [14,17]. As shown in Equation (1), the formation of CsPbX_3_ NCs is performed with excess halide while Cs-oleate is the limiting reagent. Prior to separation and purification, besides CsPbX_3_ NCs, the byproducts of lead oleate and oleylammonium halide and unreacted OA and OAm coexist in the reaction system, and act as surface binding components. Due to the nature of ionic CsPbX_3_ NCs, even more ionic interactions with capping ligands can result in the easy dissociation of binding ligands from the surface of PNCs during separation and purification [20]. Some reports show that, by adding small excess of OA and OAm before precipitating PNCs, the photoluminescence quantum yield (PLQY) and the colloidal integrity of NCs can be effectively preserved for their improved stability [14,20]. In addition, the variety of aliphatic carboxylate and amine ligand pairs can modify the morphological dimensionality and size of PNCs. It has been observed that the increase in nanocube size corresponds a decrease in acid chain lengths ranging from acetic to oleic acid and hexyl- to oleylamine, respectively (Figure 1a) [19]. Meanwhile, shorter alkyl-amines and OA can break the inherent cubic crystal structure and induce the 2D growth of PNCs, i.e., nanosheets or nanoplatelets [21]. The thickness of nanoplatelets shows an amine chain-length dependence. Pan et al., reported the thinnest nanoplatelet of 1.8 nm, obtained by using hexylamine [19]. Furthermore, as another key parameter, the reaction medium such as ether solvents (e.g., ethylene glycol dibutyl ether (EGDBE), diethylene glycol dibutyl ether (di-EGDBE), and tetraethylene glycol dibutyl ether (tetra-EGDBE)) can also affect the nucleation and growth of CsPbX_3_ NCs due to their different polarity at different temperatures (Figure 1b,c) [22]. Li et al., reported that the size and morphology of CsPbBr_3_ QDs can be well tuned into the solvent with low polarity, which also relates to the stability of as-synthesized PNCs [22].

Lead-free PNCs based on tin (Sn), CsSnX_3_, with a size of ~10 nm can be readily obtained within the hot-injection synthesis by using SnX_2_ as the metal halide source instead of PbX_2_ [24]. However, due to the easy oxidation of Sn^2+^ to Sn^4+^ in ambient conditions, the stability of as-prepared CsSnX_3_ PNCs is extremely low in air, which becomes a critical issue for their application. Alternatively, by employing SnI_4_ as a precursor within the same hot-injection synthesis, much more air-stable Cs_2_SnI_6_ PNCs can be achieved [25]. Interestingly, the reaction time ranging from 1 min to 1 h for the initial Cs_2_SnI_6_ PNCs induces the morphology transition from 1D nanorods/nanowires to 2D nanoplatelets. Some other metal substitutions, i.e., bismuth (Bi) or antimony (Sb), have been investigated towards achieving lead-free stable PNCs via the hot-injection procedure. However, those Bi- or Sb-based PNCs generally show relatively low PLQY (e.g., <5%), mainly due to their large size and different phase or deep faults originating from their electronic orbitals (e.g., Sb 5p-orbital splitting) [26,27].

Currently, as the most widely employed method for the synthesis of PNCs, the high temperature hot-injection procedure is a very promising route to obtain high-quality PNCs. Due to the dissociation of the nucleation and growth stages within the hot-injection synthetic route, a high level of monodispersity can be realized with no need of further size-selective skills [28]. However, the stability optimization of PNCs at the stage of reaction is limited, and depends to a large extent on the separation and purification of post-reaction system and post-synthesis treatments [29], which will be discussed in Section 2.2.

#### 2.1.2. Ligand Assisted Reprecipitation (LARP) Method

One critical issue of the hot-injection method is that the precise reaction temperature is hard to control due to the injection of cold solution (e.g., Cs-oleate precursor), causing low reproducibility from batch to batch and inhibiting large-scale manufacturing [30]. Therefore, some other alternative synthesis methods have been developed to tackle these problems. The low temperature ligand assisted reprecipitation method (LARP) is a representative strategy to be readily scaled up, while obtaining high production yield of stable PNCs [31,32].

Prior to the formal establishment of the LARP method, a so-called “non-template” (NT) method was initially developed to synthesize both hybrid (e.g., methylammonium(MA)PbBr_3_ PNCs) [33] and fully inorganic (e.g., CsPbBr_3_ QDs) [34] PNCs in large-scale mode. In a typical NT synthesis, the soluble precursor salts (e.g., MAX or CsX, and PbX_2_) dissolved in a DMF solution are mixed with a ligand solution which contains OA and alkyl-chain amine in ODE at low temperature (<100 °C). By adding an anti-solvent such as acetone in the mixture precursor, the PNCs can be precipitated due to the reduced solubility. A very high PLQY of >90% and large volume (up to 900 mL) of PNCs per batch can be achieved via NT synthetic route [35]. As a derivative of the NT method, Zhang et al., initially developed the LARP method to fabricate MAPbX_3_ QDs at room temperature (RT) [36]. Instead of the separated ligand solution in the NT method, all precursor salts and ligands were dissolved in a polar solvent such as DMF or DMSO. With the addition of an anti-solvent (e.g., toluene), the precipitation of MAPbBr_3_ NCs with a size of 3.3 nm was initiated, showing a PLQY of ~70%. The synthesis of functional cubic CsPbI_3_ PNCs via the LARP method has been challenged by employing CsX as Cs source due to the large particle sizes in colloidal route, reducing the phase instability [31,32]. Alternatively, by using Cs-oleate precursors, a controllable small size (~5 nm) of CsPbI_3_ QDs can be obtained while stabilizing the cubic phase [37]. As another key parameter in the LARP method, the DMF reaction method can seriously degrade and even dissolve as-prepared PNCs, resulting in a reduced production yield [36]. A homogeneous reaction process at RT has been developed by Wei et al., for synthesizing CsPbBr_3_ QDs in a mode of gram-scale production under ambient condition [32]. A series of non-polar solvents (e.g., chloroform, dichloromethane, hexane or toluene) were used to mix all Cs^+^, Pb^2+^ and Br^−^ precursors. Eventually, they found that fatty acids, i.e., long-chain carboxylate acid, such as butyric acid, hexanoic acid, octanoic acid or OA, play a significant role in the RT homogeneous synthetic route to CsPbBr_3_ NCs [14,32].

The LARP method is also suitable to synthesize lead-free and fully inorganic PNCs. Cs_3_Bi_2_X_9_ QDs have been successfully synthesized by injecting a DMSO solution consisting of CsX and BiX_3_ precursors into 2-propanol (IPA) as an anti-solvent [23]. Interestingly, without any capping ligands in the reaction, 6 nm-size Cs_3_Bi_2_Br_9_ QDs can still be formed but showing a low PLQY of 0.2%. After involving some aliphatic ligands such as OA and octylamine, smaller size QDs (e.g., ~3.9 nm) were obtained with a largely improved PLQY of 19.4% [38]. By substituting Bi with Sb at B site of their ABX_3_ structure and using a similar synthetic route, Cs_3_Sb_2_Br_9_ QDs in size of 3.1 nm were also fabricated with a dramatically enhanced PLQY of 46% [39]. All these fully inorganic PNCs showed improved thermal and chemical stabilities compared to their hybrid analogues based on storage-time dependent X-ray diffraction (XRD) study (Figure 1d) [23,38]. The main drawback of the LARP method is that it is hard to precisely control PNCs size and morphology, which can be instead easily achieved via the hot-injection method. Besides the above-discussed colloidal injection synthetic routes, non-injection methods such as convection, solvothermal, microwave, and ultrasonication methods, have also been developed for the preparation of stable metal halide PNCs. In this review, we will not discuss those non-injection methods further, but an interest reader can refer to [14,40] for a deep overview.

### 2.2. Post-Synthesis Modification

To be competitive with the high performance of bulk perovskite solar cells, it is crucial to understand the degradation mechanism of current metal halide PNCs particularly at post-synthesis stage, and then further improve the stability of PNCs for potential commercialization. Recently, several significant post-synthetic treatments have been investigated and developed to not only tune the structural and optical properties of PNCs but also to increase their stability in both solution and solid phases [41,42]. These treatments, that are closely in relation to the surface chemistry of colloidal PNCs, can be classified according to three criteria: surface passivation, phase transformations, and ions exchange, including cation exchange and anion exchange.

#### 2.2.1. Surface Passivation

**Figure 2:**
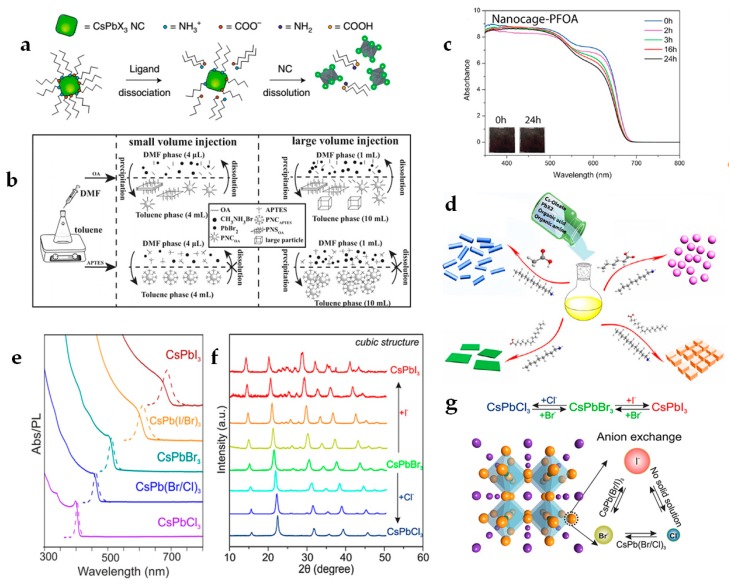
(**a**) Structural lability of CsPbX_3_ nanocrystals (NCs) due to the desorption of weakly bound ligands. Reproduced with permission [43]; (**b**) Precipitation quantities influenced by oleic acid (OA) and (3-amino-propyl)triethoxysilane (APTES) ligand capping effects. Reproduced with permission [48]; (**c**) Comparison of absorption spectra of CsSnBr_3_ nanocages after treatment with perfluorooctonoic acid (PFOA) at different exposure time. Inset: films were prepared by drop-casting the solution on a glass slide and exposure in air (25 °C, RH 60%). Reproduced with permission [50]; (**d**) Schematic illustrating the formation process for different CsPbX_3_ (X = Cl, Br, I) nanocrystals mediated by organic acid and amine ligands at room temperature. Reproduced with permission [37]; (**e**) Typical optical absorption and PL spectra of CsPbX_3_ NCs after post-synthetic anion exchange reactions. Reproduced with permission [17]; (**f**) XRD patterns of the parent CsPbBr_3_ NCs and anion-exchanged samples in cubic phase. The top pattern for purely CsPbI_3_ NCs shows some secondary phase (e.g., orthorhombic) in addition to the parent cubic phase; (**g**) Schematic of the anion-exchange within the cubic perovskite crystal structure of CsPbX_3_. Reproduced with permission [55].

Owing to the strong ionic character of both internal and external bondings, PNCs are readily formed while it is also easy to break them during the isolation and purification steps (Figure 2a) [43]. The surfactants for surface passivation, i.e., capping ligands, are therefore the key to preserve the structure of PNCs and further influence their stability and PLQY. Since straight-chain aliphatic ligands with a terminal group of –NH_2_/NH_3_^+^, i.e., oleylamine and octylamine, are used to dissolve B-site metal cation in the precursor during the synthesis, it is reasonable for amino capping ligands to originally passivate the surface of the as-formed PNCs. Due to the modest constants of ligands binding to the surface of PNCs, the ligand density at the surface typically decreases after several required cycles of isolation and purification [44]. It has been reported that, by adding a small amount of organic acid and amine ligands, the acid-base equilibrium can be maintained as a prerequisite to achieve good colloidal stability [45,46]. However, the excess of amine has also been proved to destabilize the PNCs into an irreversible degradation with a transition shown by Equation (2) [47]:

(2) $$4{\mathrm{CsPbBr}}_{3}\;\overset{\mathrm{amine}}{\to }\;{\mathrm{Cs}}_{4}{\mathrm{PbBr}}_{6}\;+\;3{\mathrm{PbBr}}_{2}$$

Instead of straight-chain ligands, Li et al., developed a passivation mechanism in terms of a so-called dissolution-precipitation model (shown in Figure 2b) by employing branched capping ligands, i.e., (3-aminopropyl) triethoxysilane (shortened as APTES) [48]. The re-dissolution of the as-synthesized PNCs in DMF solvent can be effectively hindered upon the strong steric hindrance and the silica formation through the function of APTES hydrolysis, which is favorable for maintaining their original properties and improving the stability.

Compared to those conventional alkyl amino and acid ligands, other surface agents containing phosphoric or fluoro group can also be beneficial for the enhancement of both stability and PLQY of PNCs. Shen et al., found that trioctylphosphine (TOP) can interact with PbI_2_, achieving a stable and quite reactive PbI_2_-based precursor. As-treated CsPbI_3_ QDs showed nearly 100% PLQY and improved chemical stability [49]. Deng et al., demonstrated novel lead-free hollow nanocrystals capped with perfluorooctonoic acid (PFOA) after the ligand exchange, i.e., CsSnBr_3_ cubic nanocages, exhibiting excellent oxygen resistibility and high stability against moisture and light in ambient conditions (Figure 2c) [50]. They attributed this highly improved stability to two effects from used PFOA ligands that contain strong electron-withdrawing groups with large steric hindrance.

Considering those long-chain aliphatic ligands with insulating nature, short-chain ligand exchange procedure particularly in solid state has been proposed and investigated to achieve efficient charge transfer into the device application. It has been known that PNCs are unstable in most polar anti-solvents, which makes the selection of a proper solvent for the ligand exchange in film state difficult. Short aromatic ligands such as benzoic acid and 4-phenylbutylamine recently have been able to induce the precipitation of PNCs in a suitable solvent, i.e., benzene or octane, while removing the long-chain aliphatic ligands on the surface of as-formed PNCs [51]. Pan et al., demonstrated enhanced stability of CsPbI_3_ NCs in the desired cubic phase with the surface treatment of 2,2′-iminodibenzoic acid (IDA), meanwhile exhibiting a high PLQY of ~95% [52]. In addition to conventional organic surfactant ligands, inorganic ligands could also perform as capping ligands to stabilize PNCs. For instance, with PbSO_4_-oleate capping, Kamat et al., found that as-synthesized CsPbX_3_ PNCs can be effectively protected to preserve their native structure for several days, which can restrain the reactions of anion exchange [53]. Potassium-oleate (K-oleate) has also been employed to passivate the surface of PNCs, resulting in enhanced photo- and thermal-stability [54]. Further exploitation of effective capping ligands during post-synthesis modification will be crucial to obtain highly stable and optimal optoelectronic properties of PNCs.

#### 2.2.2. Phase Transition

During the post-synthesis treatment, not only the surface of as-formed PNCs is effectively modified and passivated by capping ligands, but also the phase transition of PNCs can be induced, which is generally accompanied with the change of morphologies (0D–3D). The phase identity and phase transformation are thus the key factors to evaluate the phase stability of PNCs. For the native formation of PNCs in terms of synthesis procedure, the classic micellar transition mechanism can be the dominant scheme to shape the PNCs upon various organic acid and amine ligands [37]. One typical example of phase transformation is 3D CsPbBr_3_ nanocubes converting into 2D CsPb_2_Br_5_ nanosheets, triggered by either PbBr_2_-rich conditions (CsPbBr_3_ + PbBr_2_ → CsPb_2_Br_5_) [56,57] or by breaking down the initial 3D PNCs with ligands (2CsPbBr_3_ → CsPb_2_Br_5_ + CsBr) [58]. Several groups have found a direct transition from 3D CsPbBr_3_ to 0D Cs_4_PbBr_6_ by adding the excess of amines to extract PbBr_2_ from the parent PNCs [59,60,61]. As-transformed 0D Cs_4_PbBr_6_ PNCs showed improved size homogeneity and chemical stability with the addition of alkyl-thiol ligands [62]. Deng et al., systematically investigated the relationship between the type and length of organic acids and amines and the formed PNCs in variable phases and morphologies [37]. For instance, hexanoic acid and octylamine can modify the PNCs into 0D QDs while the oleic acid and octylamine are able to shape the PNCs into few-unit-cell-thick nanosheets (Figure 2d) [37]. Other factors such as heat [63,64], light [65], and moisture [66,67] can also trigger the phase transition of PNCs into the solid state. The transformation of Cs_4_PbBr_6_ → CsPbBr_3_ can be achieved via thermal annealing by physically removing CsBr [63]. Further exploration of multi-factor driving phase transformation during post-synthesis process will be still required to obtain optimized and phase-stable properties of PNCs.

#### 2.2.3. Ions (Anion and Cation) Exchange

It is known that the optical properties of PNCs can be tuned in the spectrum ranging from blue to red light by varying the halide composition. Due to its highly ionic character, PNCs undergo a post-synthetic anion exchange reaction (Cl^−^ ↔ Br^−^ and Br^−^ ↔ I^−^) (Figure 2e) [17]. By adding the halide source, such as PbX_2_ solution or an ammonium halide solution, a swift halide exchange can be achieved because of the precise cationic sub-lattice and the high diffusivity of halide vacancies [55]. The monodispersity of colloidal PNCs can be preserved during the anion exchange process, and it exhibits similar optical properties as those of directly formed PNCs with the same composition [55,68]. Most importantly, the phase feature of PNCs, i.e., morphology and shape, can be independently maintained, which has been proved with 1D nanowires- and 2D nanoplatelets-shaped PNCs [34,69]. Kovalenko et al., found that the composition modification of CsPbX_3_ PNCs with mixed halide anions show the limited influence on the change of the cationic sub-lattice, and the cubic phase is still effectively retained after the anion exchange reaction (Figure 2f,g) with an exceptional case that the purely CsPbI_3_ PNCs also exhibits a secondary phase (e.g., orthorhombic) in addition to the parent cubic phase [55]. However, Yang et al., demonstrated that the phase transformation can occur from cubic to orthorhombic by increasing the ratio of I^−^ anions in CsPbBr_3−*x*_I*_x_* PNCs composition [70]. This contradiction indicates that further experimental investigations and deep understanding of structure-composition relationships for PNCs are relatively urgent to better clarify the phase identity issue upon the halide anion exchange process.

As an analogous process to anion exchange, the cation exchange reaction on both the A-site and B-site of PNCs ABX_3_ structure (see Section 3) can also effectively tune and improve the optical properties and the stability of PNCs. It has been known that A-site cations weakly influence the band gaps of PNCs due to their very limited contributions to the density of states near the Fermi level [13]. Nevertheless, the symmetry of the PNCs compounds can be effectively determined by the character of A-site cations. Several reports demonstrated that for CsPbX_3_ PNCs, A-site Cs^+^ (1.67 Å) can be replaced by the larger MA^+^ (2.17 Å) cation [71], while Cs^+^ and FA^+^ (2.53 Å) can be cross-exchanged to achieve Cs_1−x_FA_x_PbI_3_ PNCs [72]. Manna et al., have reported that exchanging the A-site cations in PNCs can result in a variation in the tolerance factor and accordingly in the stability of PNCs [13]. For instance, the partial or complete exchange of Cs^+^ in CsPbI_3_ PNCs by FA^+^ cations, using FA-oleate as a precursor, leads to a long-term stability and a near-IR emission of resulting PNCs [72]. The first report on the partial exchange of the B-site Pb^2+^ cation in CsPbBr_3_ PNCs was published by Van der Stam at al., in which they mixed as-formed PNCs solution and M^2+^ cation precursors in toluene, i.e., M = Sn^2+^, Zn^2+^ and Cd^2+^ [73]. However, this method led to only limited (10%) substitution of the M^2+^ cation exchanged into the native PNCs, although a large excess of M^2+^ was applied. This observation can be attributed to the required higher activation energies at the B-site compared to anion exchange at X-site [55]. Alternatively, Mondal et al., employed the ultrasonication method to improve the alloying level (up to 40%) of CsPbCl_3_ PNCs with Cd^2+^ by using a saturated CdCl_2_ solution in ethanol [74]. The increased doping level of Cd^2+^ in Cd:CsPbCl_3_ PNCs enhanced their photo-stability while keeping the PLQY up to 90% of the initial value after several months of storage. Post-synthetic Mn^2+^ doping in CsPbX_3_ PNCs has also been employed for the cation exchange at B-site. Huang et al., employed a simultaneous halide exchange method by adding MnCl_2_ solution into as-synthesized CsPbBr_3_ PNCs to alloy the sensitized Mn:CsPbCl_3−*x*_Br*_x_* [75]. However, those alloyed PNCs showed an unstable feature from photo-oxidation and thermo-degradation. To overcome this stability issue, Xu et al., prepared CsPbMn_1−*x*_Cl*_x_* by embedding as-formed CsPbBr_3_ PNCs in a mixture of MnCl_2_ and KCl matrix for a simultaneous cation and anion exchange [76]. With this approach, several unique phenomena were observed, including the formation of intermediate core-shell structures and composition-dependent doping processes upon long reaction times (e.g., >40 h).

Additional applied strategies on cation exchange had negative effects on stabilizing the original PNCs, as in the case of the decomposition of native CsPbX_3_ PNCs upon the substitutions of Cs^+^ cations by Rb^+^, Ag^+^ or Cu^+^ at A-site, and of Pb^2+^ cations by Ge^2+^ or Bi^3+^ at B-site [40,55]. Several possible factors can affect the decomposition, including incompatible ion sizes, oxidation induced instability, or thermodynamics dependent phase competition [10]. Therefore, it is necessary to further explore and optimize the techniques of post-synthetic ions exchange to acquire stable phases of PNCs with tunable optoelectronic properties.

## 3. Perovskite Nanocrystals Structure

Metal halide perovskites have a general crystal structure of ABX_3_, in common with calcium titanium oxide (CaTiO_3_) mineral discovered in 1839 by the mineralogist Lev Perovskite, and after him named as ‘perovskite’. In ABX_3_, X is anion while A and B are cations, A being larger than B. The A cation occupies a cubo-octahedral site coordinated with twelve X anions while the B cation is stabilized in an octahedral site coordinated with six X anions.

### 3.1. A-Cation

Due to the large cavity between the octahedra in ABX_3_ structure of PNCs [14], A-site can be occupied by a single type of cations, typically Cs^+^, MA^+^, and/or formamidinium (FA)^+^, or by a mixture of them. A-site engineering offers multiple possibilities for fine-tuning structure-property-stability relationships within PNCs. If the large Pb^2+^ or Sn^2+^ cation (most common choices for B cation) occupies the B-site, the A-site cation should be large enough to meet the structural requirements predicted by the tolerance factor [15]. For PNCs, there are three most common 3D polymorphs at ambient conditions, i.e., cubic, tetragonal, and orthorhombic phases, similarly as for the bulk perovskite crystals. The relative stabilities of the different polymorphs in PNCs can be modified by surface chemistry effects [15]. Since MA^+^ is volatile, it may be desirable to avoid it in PNCs composition for ensuring long-term stability [77]. Two MA-free PNCs, FAPbI_3_ and CsPbI_3_, have recently attracted wide attention for their near-infrared emission properties. However, both of those MA-free PNCs are not highly stable due to the suboptimal A-site cations, i.e., too large FA^+^ and too small Cs^+^, which is defined as “perovskite red wall” [15,72]. To address this issue, an effective way is to synthesize a mixture of A-cations based PNCs such as Cs_1−*x*_FA*_x_*PbX_3_. Protesescu et al., demonstrated a modified hot-injection method for mixing FA^+^ into CsPbI_3_ PNCs by simply injecting FA-oleate solution together with Cs-oleate during synthesis [72]. As-synthesized FA_0.1_Cs_0.9_PbI_3_ PNCs showed a relatively high PLQY (>70%). Their cubic phase, stable for several months in ambient conditions, was responsible for the retention of their initial PLQY in a colloidal state (still more than 70% after a few months storage) [72]. Recently, Rubidium (Rb) or Potassium (K) have also been considered as alternative A-cations for all-inorganic PNCs with improved stability [77,78]. Lee et al., synthesized orthorhombic RbPbI_3_ nanowires for the first time, by simply replacing Cs_2_CO_3_ with Rb_2_CO_3_ upon the typical hot-injection method [79]. However, the tolerance factor of RbPbX_3_ (X = Br or Cl) NCs is ~0.8, which implies a limitation in perovskite formation [80]. To solve this problem, by mixing A-site cations, the tolerance factor of as-synthesized Rb_1−*x*_Cs*_x_*PbX_3_ PNCs can be effectively enhanced. However, these PNCs still exhibited comparable PLQYs to that of CsPbX_3_ NCs [40]. In addition, tuning the A-site with larger cations reduces the morphological dimensionality of PNCs to 2D sheets or platelets, 1D rods or wires, or 0D dots or clusters [81]. The morphological dimensionality related stability of PNCs will be discussed in detail in Section 3.5.

### 3.2. B-Cation: Lead-Free PNCs

The crystal structure of PNCs is mostly affected by the choice of B cation. When B in ABX_3_ is Pb, depending on the tolerance factor the perovskite structure can e.g., be cubic, consisting of a three-dimensional (3D) network of corner-sharing [PbX_6_]^4−^ octahedra, with Pb^2+^ cations located at the center of the octahedra, and with the large cavity between adjacent octahedra occupied by A^+^ cations. When Pb is replaced by other elements, for example those of group-14 in the periodic table like Sn and germanium (Ge), adjacent kin elements like Sb and Bi, or double elements in the so-called double perovskites, a great variety of crystal structures for PNCs can be obtained [82].

The B cation of the ABX_3_ perovskite structure is also the main responsible of the perovskite toxicity, a major critical problem of traditional halide perovskites with B = Pb^2+^ that makes this technology still not ready for commercialization [83]. In fact, the presence of hazardous heavy metals (like lead) in electronics is regulated in the European Union, and similar restrictions are expected to be reinforced in other countries as well. This has recently driven an intense research worldwide toward environmentally benign Pb-free perovskite materials, both in bulk and as nanocrystals, for different applications [82,84]. The development of chemically robust Pb-free halide PNCs alternatives with similar outstanding characteristics as the original lead halide PNCs has, however, so far led to materials with significantly lower performances in optoelectronic devices with respect to those based on Pb-containing analogues [85,86,87]. Therefore, it is paramount to identify new synthetically accessible Pb-free materials (both in bulk or in nanocrystal phase) that are environmentally stable and friendly while retaining the outstanding properties of Pb halide perovskites to allow perovskite technology entering the consumer electronics market [43].

While many reports exist on lead-free bulk perovskites, only a handful of them refer to lead-free perovskite nanocrystals, each focusing on different aspects [12,82,88,89]. In this Section, we look at available Pb-free PNCs systems from stability point of view. As follows, we report the key elements employed to replace Pb when aiming at perovskites with lower toxicity. When selecting elements to form Pb-free perovskite structures, several chemical design rules, both structural and electronic, can guide us to screen potential candidates [90]. As for the structural criteria, Goldschmidt tolerance (*t*) and octahedral (µ) factors, together with the cation/anion radius ratio can help to predict stable perovskite phases. It is important to note that some phases not stable in bulk are only accessible at nanoscale. One well-known Pb-based case is the α-CsPbI_3_ phase [89]. As for the electronic criteria, compositions resulting in PNCs with direct band gap and strongly allowed lowest optical transition for efficient absorption and emission processes are required for materials with good optoelectronic behavior [90]. Nanocrystals offer a wide composition flexibility, often serving as model systems to achieve compositions impossible to achieve in any other way thanks to the cation exchange, alloying, or doping. Furthermore, quantum confinement effects also affect the electronic structure and thus the optical transitions of the perovskites, thus possibly tuning their properties for more efficient optoelectronic devices [89].

The key examples of Pb-free PNCs are summarized in Table 1.

Sn and Ge halide perovskites are the structurally and electronically closest to Pb halide counterparts. The most promising alternative to Pb is Sn because of its efficient absorption and emission, suitable bandgaps with direct nature and narrower than their Pb analogues, low exciton binding energies, long carrier diffusion lengths, and defect tolerance [50,101]. To date, the highest efficiency for Pb-free perovskite solar cells is obtained with Sn-based perovskites [102]. However, Sn-based PNCs have debatable toxicity [103], and are also more unstable than the lead counterparts towards moisture, light, and heat. A unique example of Sn-based PNCs with outstanding stability against moisture, oxygen, and light are hollow CsSnBr_3_ nanocages treated with perfluorooctanoic acid, reported by Wang et al. [50]. This work demonstrates the already underlined importance of selecting and fine-tuning the synthetic methods when aiming at stable yet highly performing PNCs.

Ge is fairly rare and pricey element, which is also poorly stable [6]. Hence, Sn or Ge are not the best substitutions for Pb at B-site when targeting high stability. However Padture et al., reported a brilliant example of surface passivation with a Sn-Ge bulk perovskite, which induces the formation of an ultrathin (~5 nm) native-oxide layer on the bulk perovskite surface, responsible of a significant enhancement in the stability [104]. Inspired by this work, very recently we have successfully pioneered the synthesis of CsSn_0.5_Ge_0.5_I_3_ nanocubes with improved stability compared to CsSnI_3_ NCs in ambient conditions, as the result of the reduction of high trapping density Sn vacancies upon partial replacement of Sn with Ge atoms in the nanostructures [105].

Cations from group 15 elements, Bi^3+^ and Sb^3+^, form 1D perovskite crystals with formula A_3_B_2_X_9_, namely Cs_3_Bi_2_I_9_ and Cs_3_Sb_2_I_9_. However, reducing the morphological dimensionality from 3D to 1D increases the bandgaps and leads to anisotropic properties, not ideal when considering photovoltaic applications [106,107].

Another approach to overcome the toxicity of Pb in perovskites is to design materials with A_2_B′B^″^X_6_ structure, i.e., the so-called double perovskites (DPs), where two Pb^2+^ in the ABX_3_ crystal lattice are replaced with a pair of monovalent and trivalent metal cations B′ and B″. Their structure is referred to as elpasolite. DPs are very promising materials due to the compositional flexibility of their A_2_B′B^″^X_6_ crystalline structure. Moreover, in spite their optoelectronic properties inferior to Pb or Sn perovskites, DPs are the best materials when aiming at highly stable perovskites that are also Pb-free. Theoretically, thousands of DP compositions are possible. To identify the most promising materials to be synthesized a combined computational and machine learning approach is paramount. The most promising and widely explored DPs rely on silver (Ag) and Bi, though most of the reported double PNCs have indirect bandgap, being thus not ideal for photovoltaics or light-emitting diodes applications. On the other hand, Cs_2_AgBiBr_6_ PNCs exhibited outstanding photoconversion of CO_2_ for solar fuels production, and it has also been used for water splitting applications, together with Cs_2_AgBiCl_6_, Cs_2_AgSbBr_6_, and Cs_2_AgInCl_6_ [108]. It is interesting to note that, while Cs_2_AgBiI_6_ is not stable in bulk form (single crystals or thin films) [109,110], yet it can be stable in nanostructures [111]. As far as degradation is concerned, a decrease in chemical stability from Cs_2_AgInCl_6_ to Cs_2_AgBiCl_6_ has been observed [112].

Copper (Cu) is another promising material when aiming at humidity-stable, heat-stable, and UV light-stable lead-free perovskites. A Cu-based 2D perovskite (C_6_H_5_CH_2_NH_3_)_2_CuBr_4_ [113] has been reported by Li et al., showing a low bandgap of 1.81 eV and high absorption coefficient of ∼1 × 10^5^ cm^−1^ at 539 nm, making it a suitable light-harvester for solar cells, and particularly suitable for tandem photovoltaics. Finally, Solis-Ibarra et al. [114]. proposed a stable Cu-Sb DP with direct bandgap (hence suitable for photovoltaics) of 1 eV [114].

### 3.3. Halides

**Figure 3:**
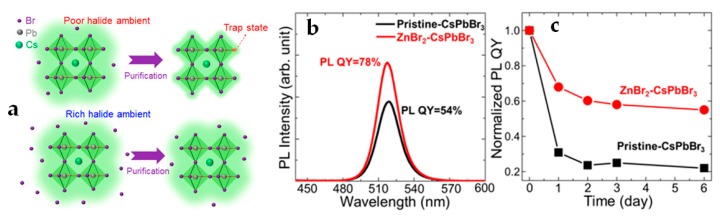
(**a**) Scheme for halide-poor and halide-rich circumstances for synthesis of PNCs. Reproduced with permission [115]. (**b**) PL spectra and (**c**) photoluminescence quantum yield (PLQY) of CsPbBr_3_ before and after self-passivation with ZnBr_2_. Reproduced with permission [116].

Halide composition engineering is recognized as a major approach to tune the structural and optical properties of both Pb-based and Pb-free PNCs. As one key parameter, the elemental ratio of halide/metal (X/B) severely influences the synthesis to obtain high quality and stable PNCs. Typically, for Pb-based PNCs, the ratio of X/Pb ranging from 2:1 to 4:1 has been verified to achieve higher PLQYs, longer PL lifetimes, and more stabilized NCs, as reported by Wang et al. [115]. The enhancement of overall optoelectronic properties upon the above-mentioned halide-rich synthesis could be attributed to the reduced traps on the surface halide vacancies of as-formed PNCs [115]. The same authors proposed a scheme of CsPbBr_3_ PNCs synthesis in halide-poor and halide-rich circumstances (Figure 3a) [115]. Due to the ligands easily peeled off from the surface of PNCs during the purification in a halide-poor circumstance, the PLQY of as-formed PNCs was dramatically decreased. On contrast, by adding more amount of oleylammonium bromide that surrounds the PNCs, the halide can effectively remain on the surface of PNCs, preserving the condition for self-passivation. The Br-rich capping can occupy the surface vacancies while passivating the surface where the electron traps allocate, resulting in improved structural stability and PLQY of CsPbBr_3_ PNCs [15]. In addition, the durability of PNCs during the purification and device manufacturing can be enhanced in the halide-rich stoichiometry [13]. Woo et al., used inorganic ZnBr_2_ as an additional source of bromide into the PbBr_2_ precursor for synthesis [116]. The resulting PNCs showed more halide-rich stoichiometric compositions (Cs:Pb:Br = 1.0:1.2:3.4) than that produced from the standard synthetic method without metal bromide (Cs:Pb:Br = 1.0:1.0:2.8). With this so-called “inorganic passivation”, the stability and initial PLQY of as-synthesized CsPbBr_3_ PNCs were considerably improved (Figure 3b,c) [116]. Instead of PbX_2_ as the standard halide source, Imran et al., employed benzoyl halides to synthesize a whole family of fully inorganic and hybrid PNCs, i.e., CsPbX_3_, FAPbX_3_, and MAPbX_3_, demonstrating a good phase purity and stability with the precise size control [117]. With a similar concept, Creutz et al., utilized silyl halides as the halide sources to synthesize the Pb-free DP Cs_2_AgBiX_6_, resulting in highly stable PNCs specifically tuned for a halide-rich ambient [118].

### 3.4. Role of Dopants in Degradation

**Figure 4:**
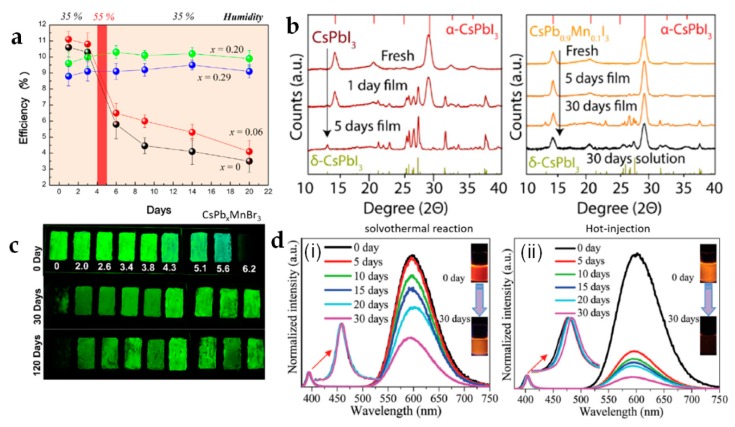
(**a**) Power conversion efficiency variation of the heterojunction solar cells based on MAPb(I_1−*x*_Br*_x_*)_3_ (*x* = 0, 0.06, 0.20, 0.29) with time stored in air at room temperature without encapsulation. Reproduced with permission [119]; (**b**) Stability of CsPbBr_3_ PNCs and CsPb_x_Mn_1-x_Br_3_ PNCs. Reproduced with permission [120]; (**c**) PL emission photographs for CsPbBr_3_:Mn PNCs QDs coated on the surface of a glass slide with different Mn^2+^ contents from 0 to 6.2 mol % taken under UV irradiation at indicated time periods. Reproduced with permission [121]; (**d**) Variation of photoluminescence (PL) spectra of the Mn-Doped CsPbCl_3_ samples prepared by (**i**) solvothermal reaction and (**ii**) hot injection method with the extension of preservation time (0–30 days). Reproduced with permission [127].

Recent studies show that doping is an effective strategy to enhance the PNCs stability [6,91]. Seok’s group introduced Br anion into MAPbI_3_ PNCs via halide exchange, leading to the formation of MAPb(I_1−*x*_Br*_x_*)_3_ [119]. MAPb(I_1−*x*_Br*_x_*)_3_ PNCs were more stable than MAPbI_3_ counterparts. Compared to the iodide-based PNCs, the bromide-doped ones had improved stability due to the higher symmetry in the nanostructure. As a consequence, while the solar cells fabricated with the undoped PNCs had power conversion efficiency rapidly decreasing and their color changing from dark brown to yellow when exposed to the humidity over 55%, once PNCs were doped with bromide anion (for *x* > 0.2 in MAPb(I_1−*x*_Br*_x_*)_3_ composition), the stability of the corresponding devices significantly improved (Figure 4a).

In addition to anion-doping, doping at A or B site with other cations can also improve PNCs stability. Protessescu et al., doped CsPbI_3_ PNCs with FA^+^ cations. The insertion of approximately 10% FA^+^ cations into the CsPbI_3_ PNCs marginally affected the cell parameters and did not change the relative intensities of the X-ray diffraction peaks [72]. As a result of the doping, the FA_0.1_Cs_0.9_PbI_3_ PNCs structure had prolonged stability compared to CsPbI_3_ PNCs (from several days to few months) in colloidal suspension as well as in films. The enhanced stability may partially originate from lattice expansion.

For the B site, currently the most used metal Pb is not only toxic but also responsible for the ionic labile Pb-halide bonds. To this aim, doping at Pb site with e.g., Mn^2+^ resulted in effectively increased structural PNCs stability derived from the high formation energy of Mn^2+^:CsPbX_3_ with respect to the pristine materials [120,121,122,123,124,125]. The degradation of drop-cast films of CsPbI_3_ and CsPb*_x_*Mn_1−*x*_I_3_ PNCs stored in ambient conditions was monitored by the evolution of their XRD patterns. CsPbI_3_ showed the sign of degradation from α-phase to δ-phase of CsPbI_3_ within one day and the transition was completed within 5 days, while CsPb*_x_*Mn_1−*x*_I_3_ PNC film did not exhibit any visible degradation during the first 2 weeks, and it mainly remained in the same phase after about one month of storage (Figure 4b) [120]. In solution, CsPbI_3_ totally degraded and turned white yellow with no photoluminescence (PL) after 1 month of storage even in nitrogen-filled glovebox, while the doped PNCs kept their color and strong PL after the same period under identical storage conditions. The improved stability can be attributed to the shrinkage of the lattice parameters upon alloying, with the shortening of the metal-X bonds (from 3.14 Å for Pb-I to 2.97 Å for Mn-I). Zhou et al., using different ratio of Mn^2+^ dopant in CsPbBr_3_ PNCs, synthesized CsPb*_x_*Mn_1−*x*_Br_3_ PNCs (*x* = 0%, 2.0%, 2.6%, 3.4%, 3.8%, 4.3%, 5.1%, 5.6%, 6.2%) [121]. As shown in Figure 4c, the green PL brightness of pure CsPbBr_3_ PNCs turned into non-luminescent PNCs within 30 days in ambient conditions. On contrast, much slower degradation was observed for Mn^2+^-doped PNCs, which still showed high fluorescence even after 120 days of storage. It should be noticed that the increase in Mn^2+^ doping concentration above 5.6% was detrimental to stability, thus it is important to fine-tune the optimal doping ratio when designing new nanocrystals. In addition to Mn^2+^, other metals have also been used for B-site doping, such as Sn^2+^, Zn^2+^, and Cd^2+^ [73,126].

When doping PNCs with the same anions, different synthesis approaches can result in different properties. Chen et al., prepared Mn-doping CsPbX_3_ by solvothermal reaction and hot injection approach, separately [127]. In this study, the PNCs obtained by solvothermal reaction showed quite stable even upon storage for 30 days in air, and the decay lifetime almost did not change. For the hot injection method, the relative emission substantially decreased after exposure to air, indicating that the loss of Mn^2+^ ions from the CsPbCl_3_ host with prolonged storage time was significant (Figure 4d). In addition, the decay lifetime for the sample exposed to air for 5–30 days (2.12–2.65 ms) was far longer than the one exposed to fresh air (1.31 ms). The solvothermal PNCs were more stable than those prepared by hot injection, which can be due to the former ones conducted under high-temperature and pressure environments leading strong bonding of Mn^2+^ in the ligand-field of the PNCs host.

As discussed above, each site (A, B, X) of ABX_3_ PNCs can be doped. Generally, going towards fully inorganic PNCs gives better stability perspectives compared to hybrid organic-inorganic ones. Combining the doping at different sites into the same PNCs should be explored in near future as an attempt to further enhance the PNCs stability.

### 3.5. Morphological Dimensionality of PNCs

**Figure 5:**
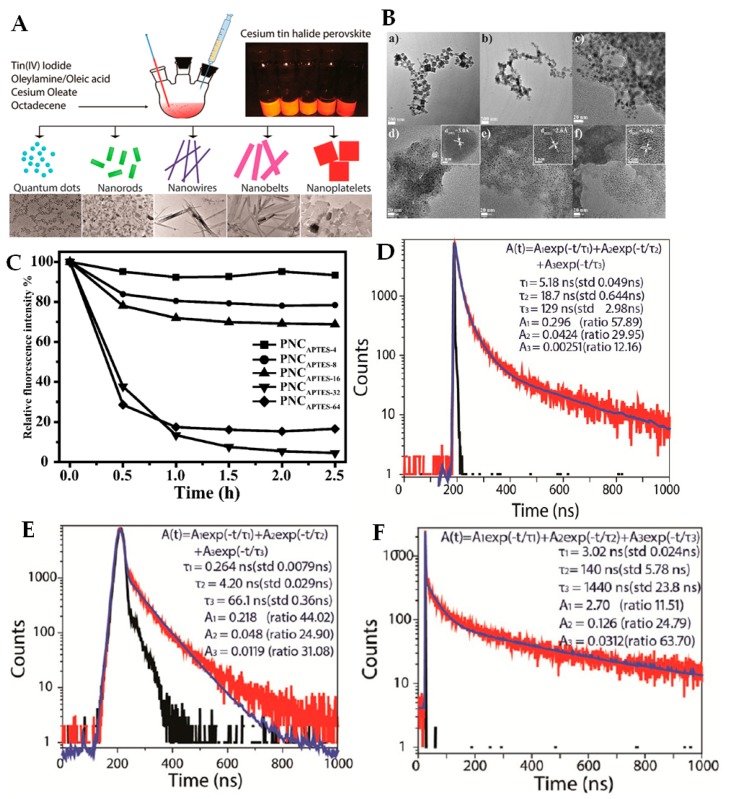
(**A**) Overview of the procedures for the synthesis of perovskite Cs_2_SnI_6_ nanocrystals with controlled morphology and TEM images of the PNCs with different shapes. Reproduced with permission [25]; (**B**) TEM images of PNCs with different sized prepared with different concentrations of APTES capping ligands; (**C**) The relative fluorescence intensity of PNC_APTES_ precipitate as a functional of time in isopropanol. Reproduced with permission [48]; (**D**–**F**) Time-resolved PL decay and fitting curves of the light emission at 515 nm (**D**, nanocubes), 514 (**E**, nanorods) or 510 (**F**, nanoplatelets) from different shaped CsPbBr_3_ NCs with a 375-pulse laser. Reproduced with permission [37].

Various chemical approaches have so far been reported to synthesize PNCs with sizes in the range from tens of micrometers to several nanometers and with variable morphologies, such as nanocubes (3D), nanoplatelets (2D), nanorods and nanowires (1D) and spherical quantum dots (0D) [6,40,128]. Using the hot-injection method, Deng’s group synthesized single-crystalline Cs_2_SnI_6_ nanocrystals with variable shapes [25]. As shown in Figure 5A, after the first 1 min of reaction, the PNCs were spherical QDs with an average diameter of ~2.5 nm, which further changed into a dominance of nanorods with length/diameter ratio of 3 nm after 5 min. Subsequently, the nanorods grew into nanowires and the length/diameter ration went up to 28 after 10 min. After 30 min, the nanowires assembled and finally formed nanobelts, which were still not stable and finally transformed into nanoplatelets with a thickness of around 8 nm after 1 h reaction. Hence, by increasing the reaction time, the size of the PNCs increased, and the shape changed from 0D to 1D and finally to 2D. This study indicates that the 2D PNCs are more morphologically stable than 1D and 0D.

Due to the surface defects and the dynamic stability of the PNCs surface, the size and morphology of the PNCs significantly influence the overall PNCs stability. Akkerman et al., fabricated 3 to 5 monolayers (MLs) of CsPbBr_3_ PNCs at room temperature, by controlling the amount of added HBr during the synthesis [34]. In this study, the stability of the 2D PNCs was related to the number of the layers: 4 MLs were only stable for one day, while 3 and 5 MLs of 2D PNCs were more stable at least for one month. Unfortunately, the reasons for the peculiar trend in stability have not been pointed out and remained unclear. However, this work demonstrates that the size of the PNCs directly influences the stability of the PNCs.

Simulation studies have shown that the surface defects may accelerate PNCs degradation [129]. Therefore, the small-sized and low dimensional PNCs are most likely the best candidates for any future application. FAPbI_3_ nanowires with tenths of micrometers in diameter exhibited phase stability up to several weeks [130]. However, once the size was reduced down to ~10 nm, the α-FAPbI_3_ PNCs with a cubic shape did not show any detectable conversion upon an extended storage after several months [72]. The higher stability of perovskites with low morphological dimensionality and their corresponding devices can be correlated to the enhanced crystallinity of the smaller clusters. Recent studies involving 1D perovskite nanowires additionally support this argument [131]. Furthermore, the results of Yong et al., regarding the small clusters of cuboids MAPbI_3_ PNCs with the higher stability compared to the large ones, also confirm the importance of low morphological dimensionality on the enhancement of stability [132].

In many cases, the small-sized PNCs showed improved photo-stability compared to the large-sized ones. However, in some cases, the conclusion reverses. Using different amounts of branched capping ligands such as (3-amino-propyl)triethoxysilane (APTES), Luo et al., obtained a tunable size of PNCs in cubic shape with an average length of 80 nm (PNC_APTES-2_), 7.8 ± 1.6 nm (PNC_APTES-8_), 5.1 ± 0.6 nm (PNC_APTES-16_), 3.1 ± 0.4 nm (PNC_APTES-32_), and 2.5 ± 0.4 nm (PNC_APTES-64_), respectively (Figure 5B) [48]. The stability of PNC_APTES_ with a larger size of PNCs was enhanced. After 2.5 h, the PNC_APTES-2_ kept almost the same PL intensity as the initial value while for PNC_APTES-6/16_ the PL intensity slightly decreased to only around 20%~30%. In contrast, the PL intensity of the small-sized PNCs, e.g., PNC _APTES-32_ and PNC_APTES-64_ with a cubic length less than 3.5 nm decreased quickly, even down to 20% of the initial value just after 1 h of storage (Figure 5C). In this work, the branched capping APTES ligands passivated the PNCs surface, and this is the key reason why the larger PNCs led to the improved stability as a result of their more effective surface passivation.

Using different ligands, Deng’s group synthesized CsPbX_3_ PNCs in various shapes, for which the long-lived lifetimes (τ_3_) of PL decays were tens to thousands of nanoseconds, and the intermediate-lifetimes (τ_2_) were several to hundreds of nanoseconds [37]. The PL decays were shape-dependent and among them the longest PL lifetime was achieved with 2D nanoplatelets (τ_3_ = 1440 ns and τ_2_ = 140 ns). For the 3D nanocubes, the τ_3_ and τ_2_ were much faster, i.e., 129 ns and 18.7 ns, respectively, while τ_3_ and τ_2_ of 2D nanorods were even more accelerated, i.e., only 66.1 ns and 4.2 ns, respectively (Figure 5D–F). Among those three shapes of PNCs, due to the atomically flat in-plane surface, the nanoplatelets had less defects and surface states than other shaped nanocrystals. The longest PL lifetime of 2D nanoplatelets can be ascribed to the increased in-plane single-crystalline geometry, with the PL decay mainly occurring via the exciton radiative recombination in the large nanocrystals. This work also suggests that the short and intermediate-lifetime can originate from trap and/or surface-state assisted PL decays.

By combining the key findings in this section, we can outline the key characteristics of stable PNCs, i.e., the small size and low morphological dimensionality, combined with good crystallinity and low defect density, and the effective passivation of the perovskite surface.

## 4. Environmental Stability (Heat, Moisture, Oxygen, UV Light) of PNCs

When the environmental stability of PNCs is targeted, i.e., the extrinsic stability towards heat, moisture, oxygen, and UV light, different approaches can be adopted depending on the specific targeted properties (e.g., resilience to heat) and applications. Several criteria in selecting one or another approach need to be evaluated, such as the cost of the materials and manufacturing process, and the environmental friendliness of the involved components.

### 4.1. Encapsulation

**Figure 6:**
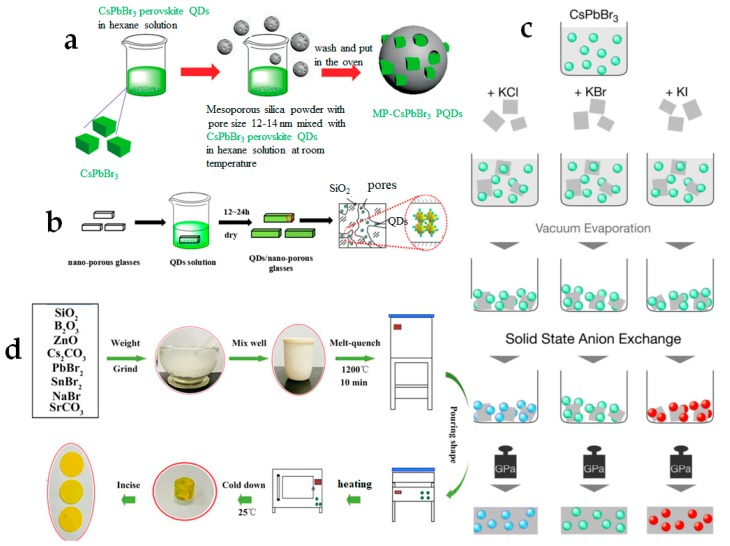
Encapsulation methods. (**a**) Integration into mesoporous particles. Modified from the original figure. Reproduced with permission [133]; (**b**) Direct embedding in ready-made porous matrix. Reproduced with permission [134]; (**c**) “cold-flow” method. Reproduced with permission [135]; (**d**) Modified from the original figure. Reproduced with permission [136].

Encapsulation is the term usually referred to the technique of incorporating nanocrystals or QDs into a protective media during the manufacturing process. The main goal is to obtain a homogeneous dispersion of emitting particles into the protective matrix to avoid agglomeration and quenching of the luminescence. The encapsulation techniques (Figure 6) adopted for producing composite materials based on PNCs can be classified into four methods, namely integration into mesoporous particles, direct embedding in ready-made solid porous matrix, cold-flow technique, and glass encapsulation.

The first approach is based on mixing the PNCs solution with ready-made mesoporous micro- and nano-sized particles (Figure 6a) [133,137,138]. While in earlier works SiO_2_-based mesoporous particles were adopted, [133,137,138] later on also attempts to use other compounds, such as Al_2_O_3_, have been reported [139]. However, the latter compounds exhibited poorer stability in comparison with SiO_2_. To date, the most promising nanocomposite in terms of stability has been synthesized by the mixture of the colloidal CsPbBr_3_ PNCs with commercially available mesoporous silica [138]. The solution is then centrifuged and dried in vacuum oven, leading to a light-emitting precipitate with strong resistance to water, UV light, and heat. When the perovskite powder was immersed in water for 120 h under UV exposure, PL was still observed. Heating to 125 °C can also still partially preserve the material. After 30 days of storage in ambient conditions, only a slight increase in PL intensity was detected.

One of the easiest ways of producing perovskite-based composites is the integration of PNCs colloidal solution into the protective solid porous media, by the direct placing of the matrix into the solution (Figure 6b). The porosity of the media allows the PNCs to penetrate into its volume and thus be more stable upon environmental exposure. However, the stability of the material remains questionable and strongly depends on the properties of the used matrix, such as the size and the volume of the porous media. Han et al. [134] prepared composite materials by soaking as-prepared borosilicate glass in PNCs colloidal solution. They obtained CsPbBr_3_/glass, CsPb(Cl_0.5_Br_0.5_)_3_/glass and CsPb(Br_0.4_I_0.6_)_3_/glass composites, whose stabilities were tested. CsPbBr_3_/glass composite showed the best performance among all composites. The sample demonstrated relatively high thermal stability (up to 280 °C) and more than 12 h of resistance to UV-light. Integration of the PNCs (CsPbX_3_, where X = Cl/Br, Br, I) into the porous opal matrices, which were grown from amorphous SiO_2_ spheres, was reported in [136]. The stability of the samples kept in ambient conditions for a month, was investigated by tracking the changes of PL peak position, PL full width at half maximum (FWHM), and average PL lifetime, respectively. All observed changes were insignificant and proved the stability of the materials. Moreover, the double and triple mixtures of PNCs with different compositions were embedded into opal matrix and no considerable change in PL occurred after a month, confirming the absence of anion exchange from the PNCs into the matrix.

Guhrenz et al. [135] showed a method based on the “cold-flow” technique (which refers to the tendency of some solid materials to deform permanently under the long-term influence of mechanical stresses) with halide salts, allowing not only to embed PNCs into protective media but also to perform anion exchange reaction for wavelength tuning (Figure 6c). The latter was realized because the matrices were used as the active components of the composites. By changing the halide component in the salt, anion-exchanged PNCs pellets were obtained, emitting at desired wavelengths. Usually, perovskite-based composites demonstrate a decrease in the PLQY in comparison to that of the PNCs in solution [140,141]. Interestingly, in this case a slight increase in PLQY from 77% to 83% for CsPbBr_3_ composites was observed. Moreover, while it is well known that PNCs lose about half of their initial PLQY upon anion exchange, [142] in this example PLQY (79%) did not decrease [135]. The obtained pellets were coated with a protective silicone layer. The stability of the silicone-encapsulated PNCs pellets under ambient condition was heavily dependent on the composition, and was preserved for at least 14 days [135]. Further investigation of “cold-flow” technique with halide PNCs is still required. Nevertheless, only one additional article has been published to date on this topic, [76] in which cation exchange between CsPbBr_3_ PNCs and a mixture of KCl and MnCl_2_ salt is demonstrated. However, in this article the stability of the proposed material has not been investigated. Moreover, probably due to Mn-doping, PLQY (0.54%) was lower than the earlier reported values.

The glass encapsulation technique during the glass synthesis is also referred to as the melting-quenching method, which is an established approach for glass manufacturing (Figure 6d). The procedure for producing perovskite glass is as follows: (1) glass ingredients as a raw material are mixed with perovskite precursors; (2) this mixture is melted at high temperature (up to 1100 °C) (at this stage perovskite precursors are in the form of ions and dispersed in glass); (3) to remove the residual thermal stress, melt portion is annealed in a muffle furnace and then cooled down; (4) samples are reheated again (at about 400 °C) for a long period of time (at this phase perovskite ions absorb energy and form nanocrystals in the glass matrix); (5) final product is cut and polished for future application. Temperatures, time, molar concentration of the compounds, and other conditions may be accordingly tuned to achieve desired properties [143]. Different glass systems have been used with PNCs such as zinc borosilicate (ZnO-SiO_2_-B_2_O_3_) [136,144,145,146,147], phosphate (P_2_O_5_-SiO_2_-ZnO) [148], (P_2_O_2_-SiO_2_-ZnO) [149], GeO_2_−B_2_O_3_−ZnO−Na_2_O [150], tellurite based glasses (TeO_2_-Al_2_O_3_-H_3_BO_3_-ZnO-Na_2_CO_3_) [151].

### 4.2. Polymer Matrix

Integration into polymer matrix consists in mixing selected polymers with a perovskite solution, containing pre-made PNCs or perovskite precursors. In the latter case, in situ formation of PNCs takes place, which is considered more efficient in terms of quality of surface passivation and dispersity of the NCs. However, the first technique is very common for composites production. For effective connection of the PNCs surface with preferred polymer, a ligand or another polymer can be introduced at the synthesis stage. The selection of the polymer is usually driven by the targeted advantages of the final material, such as flexibility, hydrophobicity, or long-term barrier properties. For further usage in photovoltaics, the obtained colloidal solution is usually cast on a surface and dried out.

Integration into polymer matrix can be used as a simple and effective way to improve the properties of the perovskite-based materials. Raja et al. [157] revealed that mixing PNCs (CsPbBr_3_) with poly(styrene-ethylene-butylene-styrene (SEBS) can significantly improve water stability for more than 122 days because of the high contact angle (or, in other words, low wettability) of this polymer. Moreover, SEBS assisted to reduce the toxicity of Pb in the composition. In fact, the amount of Pb measured after soaking the samples in water was 200 times smaller than the one contained in unprotected PNCs, reducing the concentration of Pb to the same values as those contained in drinkable tap water. This work also studied poly(lauryl methacrylate) (PLMA) performance with PNCs. While PLMA-composites were relatively stable in ambient conditions, on the other hand, they demonstrated partial solubility in aqueous solution. Huang et al., dispersed ethylcellulose (EC) in colloidal CsPb(Cl/Br)_3_ PNCs [158]. The obtained solution was spin-coated onto an optical glass. Stability was demonstrated up to 6 days in environmental conditions. A self-healing polymer matrix was introduced in [159]. Poly(dimethylsiloxane)-urea (PDMS-urea) copolymer was mixed with MAPbBr_3_ or MAPbI_3_ for producing composites. The healing ability was shown by cutting films and monitoring the scratch. The damaged area was completely recovered, and optical properties remained constant after 15 min after the incision. The healing rates could be accelerated to 5 min by heating up to 50 °C. The samples also demonstrated significant flexibility and could be stretched up to 500% of their original length.

**Figure 7:**
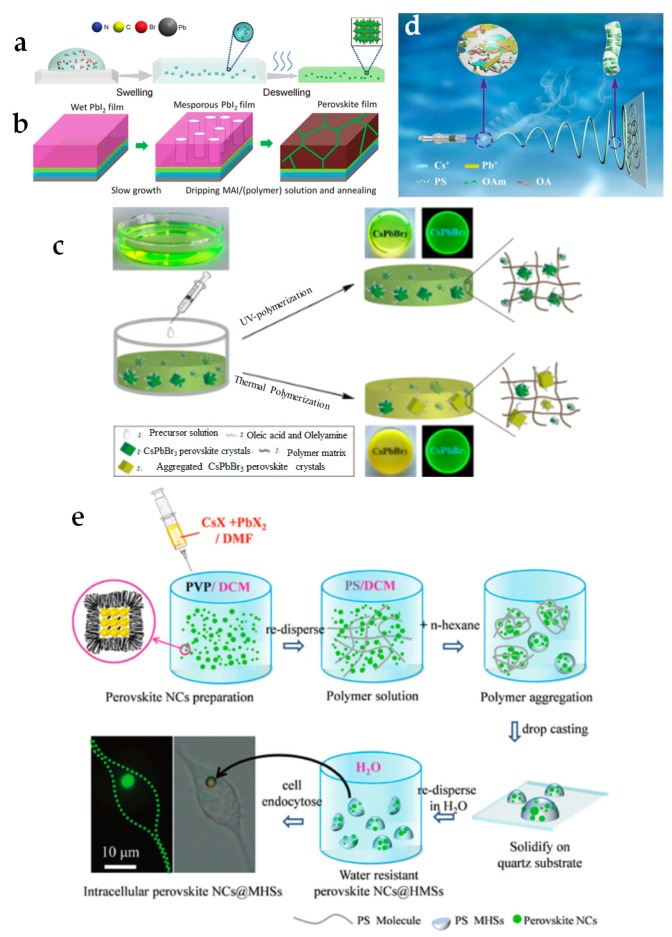
Techniques of integration into polymer matrix. (**a**) Swelling–deswelling approach. Reproduced with permission [152]; (**b**) Interdiffusion method. Reproduced with permission [153]; (**c**) UV or thermal polymerization of composite by adding initiator of polymerization. Reproduced with permission [154]; (**d**) Perovskite fiber obtained by electrospinning process. Reproduced with permission [155]; (**e**) PNCs integrated into polystyrene-based microhemispheres. Reproduced with permission [156].

In a comprehensive research by Wang et al. [152], the optical properties of several polymers such as (polystyrene (PS), polycarbonate (PC), acrylonitrile butadiene styrene (ABS), cellulose acetate (CA), poly(vinyl chloride) (PVC), and poly(methyl methacrylate) (PMMA)) were studied when combined to MAPbBr_3_ PNCs in various synthesis conditions. The polymeric films were obtained via a so-called swelling–deswelling technique. This technique enables the polymer to dissolve in a “good” solvent, where the polymer chains can expand upon the sequential solvent evaporation. During the evaporation process, the polymers shrink, leading to strong binding on the PNCs layer (Figure 7a). A set of experiments to reveal the stability of PNCs was conducted. Water treatments showed the enhanced stability of PNCs in PS, PC, ABS and PVC matrices. In fact, only a slight decrease of QY was observed after boiling the PC-based and PS-based films for 30 min. The thermal resistance of the composite generally depends on the thermal stability of the selected polymer and it is rarely measured for polymer-based composite films (Table 2). However, it was shown that PC films can withstand to temperatures up to 180 °C. Wei et al. [160] performed the same technique with pre-made PS beads and CsPbBr_3_ PNCs using toluene as a good solvent and hexane as a bad one. This method can effectively prevent anion exchange, which was proven after one week followed by the direct mixing of the PNCs/PS powders emitting blue and green light, respectively. The PS beads-based composite can survive more than 9 months in water, which is the best result achieved so far for perovskite/polymer composite (Table 2). The toxicity level of the material was also examined, by stirring the powder in water for 24 h, subsequently separating the composite, and finally drying the residual water. The amount of Pb found in the supernatant was 1.931 ppm, which was even lower than the allowed quantity for drinking water quality standards (10–15 ppm). Extremely high water stability was shown by ethylene vinyl acetate (EVA) mixed with CsPbBr_3_ precursors [161]. The authors reported that, after 720 h of storage in water, both the appearance and luminescence property of the films did not remarkably change. Moreover, the material demonstrated high flexibility by withstanding up to 1000 bending cycles with unchanged PL properties. Bright PL was detected after the composite was stretched to 350% of its original length.

In [153], the PSCs were constructed based on three different polymers, i.e., branched poly(ethyleneimine) (b-PEI), Poly(vinylpyrrolidone) (PVP), and poly(acrylic acid) (PAA) via inter-diffusion method. This approach relies on the formation of the perovskite precursor (PbI_2_) film on the substrate, followed by dripping a mixture of the chosen polymer with the second perovskite precursor (MAI) (Figure 7b). Due to the filter effect, polymer efficiently segregated at the perovskite layer and formed a homogeneous film after annealing. The authors reported a considerable enhancement of power conversion efficiency from 16.9% to 18.8% in PVP-based PSCs, which was relatively stable at ambient conditions for over 90 days.

Xin et al. [154] demonstrated the UV polymerization of the composite by addition of UV initiator (diphenyl(2,4,6-trimethylbenzoyl)phosphine oxide (TPO). This method enhanced the quantum yield of the material compared to the thermal polymerization (azobisisobutyronitrile was used as a thermal initiator) (Figure 7c). The concept was proven for PMMA and poly(butyl methacrylate) (PBMA) but it did not work for PS because of the low polymerization constant of styrene. Bagherzadeh et al. [162] embedded PNCs into a polymeric matrix based on the mixture of methyl methacrylate (MMA) and PMMA. For the initiation polymerization process, AIBN was added. The colloidal solution was poured into a casting mold and annealed to form the homogeneous plate. Because the samples were utilized as luminescent solar concentrators, resistance to UV light was investigated via continuous measurement of relative PL intensity under UV exposure (9 W). Depending on PNCs concentration, a decrease in brightness up to 25% after 12 h of excitation was reported. However, there were no significant changes in PL parameters after 24 h. Zhao et al., incorporated CsPb(Br*_x_*I_1−*x*_)_3_ perovskite QDs into the polymer matrix by dispersion of QDs into poly(lauryl methacrylate-co-ethylene glycol dimethacrylate) (PLMA-EGDA) with a UV initiator (TPO). [163] Composites demonstrated outstanding resistance to UV light—no remarkable changes in PL was observed upon four hours of strong UV illumination (100 mW/cm^2^, corresponding to 20 sun illumination). Stability under ambient conditions was confirmed up to 5 months of storage.

Another interesting work based on the swelling–deswelling technique has been reported by Liao et al., in [155]. PS was mixed with perovskite precursors and the electrospinning (ES) was utilized to cast fibers (Figure 7d). The authors also confirmed the effectiveness of this approach by synthesizing a series of perovskite films with different composition by adjusting molar ratio of precursors during manufacturing (e.g., CsPbCl_3_, CsPbCl_2_Br_1_, CsPbCl_1.5_Br_1.5_, CsPbCl_1_Br_2_, CsPbBr_3_, CsPbBr_2_I_1_, CsPbBr_1.5_I_1.5_, CsPbBr_1_I_2_, and CsPbI_3_) and applied the method also to (poly(vinyl acetate) (PVAc). The stability of CsPbX_3_/PVAc composites was studied, showing a slight decrease to 70% of the initial PLQY after 192 h of storage in water and the samples were still fluorescent after 30 days. Continuous heat at 80 °C for 120 min led to the reduction of PLQY to 50% of its initial value. The ES process for producing flexible hydrophobic fibers was also used by Lin et al., [164]. The composites were obtained by mixing PNCs (CsPbX_3_, where X = Cl, Br, and I) with poly(styrene-butadiene-styrene) (SBS). The dimensions of the fibers were controlled by variable ES parameters. The stability of the materials was investigated upon the influence of water and stretching. Depending on the chemical composition, PL vanished after keeping the samples in water for one hour. However, significant changes in PL brightness were observed for all compositions after already 10 min. RGB light-emitting diode (LED) based on green and red PNCs showed outstanding resistance to stretching (luminescence was detected after 170% of the strain). A similar approach was applied by Hai et al., using PVP instead, although the authors coated the obtained films by silicone resin for better protection. [165] Moreover, the authors synthesized three different perovskite composites (CsPbX_3_) and demonstrated that no anion exchange occurred in a three-compound mixture of them. CsPbBr_3_-based composite demonstrated resistance to water greater than 5 days, resistance to heat higher than 100 °C, and resistance to UV light longer than 120 h of radiation. Flexible LED based on the mixture of three compounds showed insignificant decrease in PL intensity after storage in ambient conditions.

Zhang et al. [156] embedded PNCs into microhemispheres (MHSs) of polystyrene matrix (Figure 7e). PVP was quickly added into a mixture of perovskite precursors to passivate the surface and to induce the micelles formation. When the mixture of PNCs and PVP was introduced into the PS solution, due to the interfacial tension force PS molecules prefer to aggregate into spherical micelles and then PNCs penetrate into them. After dropping the solution onto a quartz substrate, spherical micelles form microhemispheres for the effect of gravity force.

### 4.3. Core/Shell Structure

Shell coating is a well-established procedure that is extensively used for modifying the properties of QDs and nanocrystals. The shell can effectively prevent the interaction between nanoparticles that causes aggregation and in turn results in non-radiative energy transfer. Moreover, the passivation effect reduces the surface defects leading to trap states and non-radiative relaxation, which negatively influences the corresponding device performance. Various types of shells have been utilized with perovskite, classified into four main categories, namely oxide shell, semiconductor shell (usually another perovskite composition), polymer shell, and multilayer shell.

**Figure 8:**
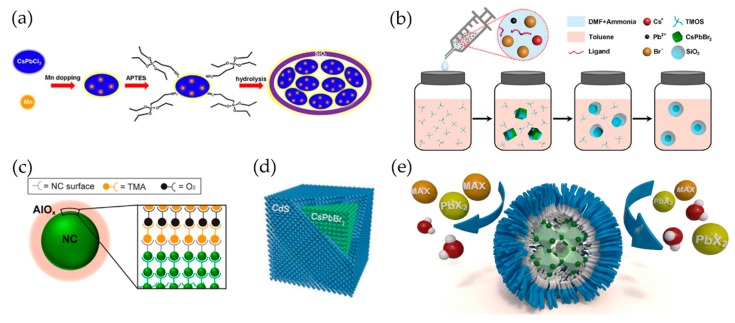
Core/shell approaches utilized with PNCs. (**a**) Silica-based core/shell perovskite particles. Reproduced with permission [166]; (**b**) Single silica-based core/shell perovskite particles. Reproduced with permission [167]; (**c**) AlO_x_ shell on top of the PNCs. Reproduced with permission [168]; (**d**) Semiconductor shell covering PNCs. Reproduced with permission [169]; (**e**) Polymer-based shell. Reproduced with permission [170].

Oxide shells are usually synthesized via hydrolysis reactions, by an approach also known as sol-gel processing [168]. The most widely applied group of precursors for this purpose are silicon alkoxides (alkoxysilanes) [171]. These precursors are injected into perovskite precursors at synthesis stage, then the mixture is continuously stirred to obtain a hydrolysis reaction. The process can be described as wrapping PNCs by the oxide shell (Figure 8a). The result is a composite in the form of gel or precipitate, which can be used further. The main drawback of this approach is the simultaneous growth of certain amount of coreless oxide particles, which are hard to separate from the core/shell PNCs.

Sun et al., obtained flexible QDs films due to the one step silanization process using APTES as the silica precursor [140]. Good encapsulation, which was proven by the absence of anion exchange reaction, led to the high resistance of materials towards detrimental exposure. The authors reported about at least one day stability of the composites after immersing them in water. The full degradation due to heat occurred at 90 °C. PL was detected after 22 days of illumination of 450 nm LED, which is a considerably longer lifetime compared to that of pure QDs. Ding et al., encapsulated CsPbX_3_ into monodisperse silica nanoplates via the modified Stöber method using tetraethyl orthosilicate (TEOS) [172]. The obtained composites were highly water-soluble and retained their PL during at least 240 h of storage in water. In ambient conditions, the coated PNCs also demonstrated high stability with practically unchanged PL up to 30 days. Obviously, multiple cores covered by one shell can be less beneficial in terms of their optical properties, leading to aggregation and anion exchange. Zhong et al. [167] demonstrated a method where a single shell covers a single core (Figure 8b). The single core/shell PNCs were obtained by a fast injection of perovskite precursor in tetramethyl orthosilicate (TMOS) (used as silica precursor) solution dissolved in a bad solvent. X-ray diffraction (XRD) analysis, performed in air upon a high humidity level (75%), confirmed the stability of the composite. To test water resistance, the material was dissolved in water by ultrasonication. Interestingly, after 16 min a clear increase in PL intensity was observed, which can be explained by the enhanced dispersity of PNCs. Even though PL started to decrease after 16 min, its intensity was still slightly higher than the initial one after 40 min. Zhang et al. [173] added ʟ-α-phosphatidylcholine (LP) surfactant and silica precursor (TMOS) into PNCs colloidal solution (CsPbBr_3_) to form core/shell composite. Multinuclear-coated nanoparticles and mononuclear-coated nanoparticles were found into the precipitate and the supernatant, respectively. Outstanding thermal, water, UV and oxidation resistances were shown by the samples, similar for both types of coating. PL intensity was not varied after the composites were heated up to 120 °C, illuminated for 168 h of UV radiation, immersed in water for 35 min, or stored for 30 days in ambient conditions.

Other types of oxide shells can be formed on top of the PNCs, too. Loiudice et al. [168] introduced PNCs (CsPbX_3_, were X = Br, I, Cl) which were coated by AlO*_x_* shells with controlled thickness (trimethylaluminum (TMA) was used as aluminium source) (Figure 8c). During the manufacturing process, the pure oxygen was applied to promote the formation of uniform and bright emitting particles. By fine-tuning the synthesis conditions and additives concentration, authors achieved an increase of the initial PLQY (75.3%). PL was still detected after more than 7 days while the particles were immersed in water. Moreover, the applied shell slowed down the anion exchange reaction and allowed studying the structural evolution of PNCs. Li et al. [174] synthesized PNCs core/shell structure by TiO_2_ coating via mixing titanium precursor (titanium butoxide (TBOT)) with colloidal CsPbBr_3_. Core/shell PNCs were formed by continuously stirring for 3 h via hydrolysis reaction followed by annealing to evaporate the excess water and organic matter. A small decrease in the PL intensity was detected after the composite was stored for more than 12 weeks in water or was irradiated with UV light for 24 h. To further investigate the stability properties, anion exchange tests were performed by blending core/shell particles with Cl and I salts. No significant changes in the PL intensity occurred after 15 days after the mixing, proving the absence of the anion exchange process and the stability enhancement of the structure.

Additional advantages can be found when other semiconductors shells cover the PNCs. Applying a shell with larger bandgap than that of the core can suppress the non-radiative recombination, contributing to the enhancement of the optical properties. Tang et al. [175] developed efficient LEDs based on a CsPbBr_3_/CdS core/shell structure (Figure 8d). However, stability analysis [169] showed a slight decrease of PL intensity after 13.5 h under high humidity level, dropping down to 40% after continuous heat up to 60 °C for 9 h. Wang and co-workers [176] proposed to use rubidium oleate (RbOA) precursor to form Rb_4_PbBr_6_ shell on top of the PNCs. The authors succeeded to increase PLQY from 40% of the pure PNCs to 85% of the composite. Photostability was tested under the illumination with UV diode (175 mW/cm^2^). In total, 90% of PLQY was kept even after 42 h, which is a much better result than the one shown by CdSe/CdS QDs measured under the same conditions.

Growing another type of perovskite derivative on top of the PNCs was recently proposed as a convenient method for enhancing their stability while simultaneously improving the optical performance. The shell could be formed by carefully adjusting the synthesis process, such as the precursor concentration, temperature, or time. One example is provided by CsPbBr_3_ PNCs covered with a CsPb_2_Br_5_ layer upon controlling the precursors’ concentration and temperature during the synthesis process [177]. Particles demonstrated relatively high PL brightness up to 5 days after being immersed in water. Jia et al. [178] showed a similar approach, forming the perovskite shell on PNCs by adding zinc bromide into perovskite solution. As a result, CsPbBr_3_/Cs_4_PbBr_6_, CsPbCl_3_/Cs_4_PbCl_6_ and CsPbI_3_/Cs_4_PbI_6_ core/shell structures were obtained. Wang et al. [179] developed an amorphous CsPbBr*_x_* shell on the top of CsPbBr_3_ through a facile hot injection technique and further centrifugation. Due to the amorphous nature of the shell, promising UV protection could be achieved. Xu et al. [180] covered Mn^2+^-doped CsPbCl_3_ by undoped CsPbCl_3_ shell. Relatively high PLQY for Mn-doped PNCs was shown (40%), and a slight increase of PL intensity was detected during the heating up to 100 °C, which can be explained by thermal annealing of defects. Bhaumik et al. [181] grew octylammonium (C_8_H_17_NH_3_, OA) led bromide 2d shell on top of MAPbBr_3_ by mixing MABr and OABr precursors. By optimizing the molar ratio of the components, they obtained perovskite nanoparticles with high PLQY (92%), stable under ambient conditions for more than 60 days. The remarkable increase in the PLQY upon semiconductor shell coating demonstrated by the above-mentioned works is a well-recognized strategy that suggests how proper band gap alignment between core and shell contributes to enhanced performance of the composite.

A polymer-based shell was also introduced as one of the routes to form core/shell composite (Figure 8e). Huang et al., formed core/shell structure by coating of β-cyclodextrin (β-CD), which is a cyclic oligosaccharide, on top of the MAPbBr_3_ [182]. Capping ligand hexylamine was introduced into the system to realize host-guest interaction reaction. Thermal stability was tested by continuous heat at 70 °C. After the first 4 h, PL intensity was increased up to 180% due to the annealing effect, which resulted in better crystallinity of the MAPbBr_3_ nanocrystals. Samples demonstrated high resistance to UV light, preserving 55% of initial PL intensity after 144 h of UV illumination. Moreover, a relatively high PL intensity was observed after immersing the composite in water for 10 days. Vijila et al. [183] mixed PNCs with PMMA solution in order to achieve PNCs covered by the polymer. The composite film was obtained by the solution casting method and dried at 60 °C. Incredible stability was shown under ambient conditions storage. After one year, 13% of PLQY was retained from the initial value of 88%. To date, this is one of the longest stability tests ever performed. Hintermayr and co-workers introduced an approach in which diblock-copolymer (polystyrene-poly(2-vinlypyridine) (PS-b-P2VP) formed micelles for protecting the PNCs core [170]. Perovskite precursors were added into micelles colloidal solution where, due to the diffusion, they penetrated the micelles and crystallized. This strategy is currently one of the best among the shell-based ones, in terms of resilience of the perovskite composite towards harmful conditions. PL intensity was observed even after 75 days of the immersing samples in water, and after more than 220 days of storage in the environment.

Simultaneous use of different types of materials to obtain multilayer core/shell structures was also investigated. Huang’s team coated MAPbBr_3_ PNCs by SiO_2_ spheres relying on hydrolysis process (TMOS was used as silica precursor), then PMMA was added into colloidal solution and thin films were formed [141]. Authors reported high resistance of the composite material against UV light (about 100 h), though in this work the samples were tested under high humidity level, therefore being not comparable with other perovskite-based composite materials reported by other researchers.

**Figure 9:**
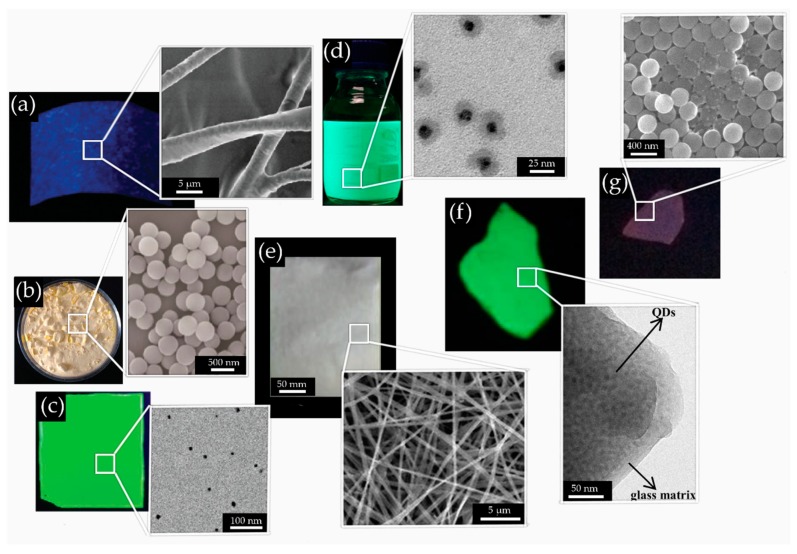
A summary of the key encapsulation methods. (**a**) Flexible silicone resin (SR)/polyvinylpyrrolidone (PVP)–based composite under UV light. In the inset, SEM images of the material. Reproduced with permission [165]; (**b**) Photo of PNCs/PS powder. In the inset, SEM image of PNCs into the PS spheres. Reproduced with permission [160]; (**c**) Photo of MAPbBr_3_/PS film excited by UV light. In the inset, cross-section TEM image. Reproduced with permission [152]; (**d**) Photo of CsPbBr_3_/SiO_2_ core-shell NCs into colloidal solution. In the inset, TEM image of the structure. Reproduced with permission [167]; (**e**) Photo of CsPbBr_3_/PS composite. In the inset, secondary electron SEM images of CsPbBr_3_/PS fibers. Reproduced with permission [155]; (**f**) Photo of CsPbBr_3_/TeO_2_-based glass under UV light. In the inset, TEM images of the structure. Reproduced with permission [151]; (**g**) Photo of PNCs into opal matrix. In the inset, SEM image of the structure. Reproduced with permission [184].

Figure 9 shows a summary of the key encapsulation methods. Table 2 summarizes the stability properties of composites in the above-mentioned works. The comparison is based on changes in PL intensity.

## 5. Conclusions and Future Outlook

Metal halide perovskites have recently emerged as the key materials for the development of highly efficient, low-cost, and printable photovoltaics (PVs). However, their poor stability, lagging well behind that of traditional PVs materials (e.g., silicon), is seriously restricting any practical utilization of this technology. Colloidal PNCs allow us exploiting the attractive optoelectronic properties of bulk metal halide perovskites with quantum confinement effects at the nanoscale. Similarly, as their bulk counterparts, PNCs also suffer from the mediocre intrinsic stability. This challenge could be addressed in many ways, including but not limited to optimizing the synthesis conditions, developing new perovskites compositions, or post-synthetic treatments. Furthermore, when exposed to environmental conditions such as heat, moisture, oxygen, and/or UV light, measures should be taken to improve PNCs’ extrinsic stability.

There are several well-established PNCs synthesis approaches, such as hot-injection and LARP. Though widely adopted worldwide, further work is needed to overcome the limited size and morphology control, the necessity of purification, and the complexity in view of large-scale manufacturing. It is expected that the optimization in the synthesis techniques will also influence the stability of the obtained nanocrystals. Due to the difficulty of the manufacturing process and poorer photovoltaic performance, Pb-free perovskite compositions are still underrepresented in the literature compared to the Pb-based ones. First works related to Pb-free PNCs have been reported only recently. However, the future development of PNCs can no longer ignore Pb toxicity. Therefore, future research should focus more intensively on establishing novel approaches for Pb-free compositions that, ideally, are also intrinsically stable. One promising direction in this context is the development of halide double perovskites, though the field is still in its early infancy and their utilization in PVs has so far resulted in devices with significantly lower efficiency compared to that of Pb-based perovskite devices. On the other hand, popular Sn^2+^ based PNCs have led so far to the best device performance among Pb-free perovskite candidates. Yet, Sn^2+^ is easily oxidized to Sn^4+^ in ambient conditions. To take advantage of the promising properties of Sn and simultaneously achieve reasonable stability, Sn^2+^ could be mixed with other air-stable divalent metal cations, such as Mg^2+^, Mn^2+^, and some other transition metals like Fe^2+^ or Zn^2+^.

Based on this review on the up-to-date multiple approaches to stabilize PNCs, we propose several key strategies to tackle PNCs degradation. First, from PNCs composition point of view, all-inorganic metal halide PNCs should be always prioritized, with accurate tuning at A-site (e.g., Cs, K, Rb) and B-site (Pb-free) metal cations towards symmetric nanostructures. As additional strategy, various surface (ligands) passivation methods should be continuously investigated for high quality and highly stable PNCs. Several properties of ideal ligands, including strong electron-withdrawing ability with steric hindrance and high hydrophobicity, [50] should be considered as selection criteria to effectively passivate the surface of PNCs against oxidization and moisture. Moreover, halogen or hydrogen bonding could be exploited as powerful strategies to passivate the PNCs surface. Since it has been found out that the surface ligands influence the stability of PNCs and their PLQYs in an opposite way, a trade-off needs to be found in order to achieve both stable and highly performing PNCs [15]. Another important strategy to effectively protect PNCs is the so-called core/shell structure. The shell materials can be either organic (e.g., polymers), [162] or inorganic or even another type of perovskite (as reported in the case of Cs_4_PbBr_6_ [178]) suggesting an alternative direction for future stability enhancement studies in this field.

Finally, a careful design of composite materials has proven to be beneficial in tackling the degradation issues by boosting the material’s resistance to ambient exposure while enhancing the optoelectronic performance at the same time. The synthesis of PNCs into glass systems or in polymer matrices, in addition to the above-mentioned core/shell structure, has been recently proposed to this aim. Despite the success in terms of stability, all these techniques require further optimization. Generally, glass encapsulation demonstrates better water stability and UV-resistance, while the polymer matrix approach is the most convenient and eco-friendly way to obtain composite PNCs structures with improved photovoltaic characteristics with respect to those of pristine PNCs. Therefore, when designing a composite material, it is essential to consider its future utilization and environmental impact.

## Figures and Tables

**Table 1:** Key examples of reported lead-free perovskite nanocrystals.

Material	Crystal Structure	Optical Transition	Bandgap, eV	Ref.
CsSnX_3_ (X = Br, Cl, I)	Cubic (Cl), orthorhombic (Br, I)	Hot injection or anion exchange	High hole mobility; Tunable bandgap through Vis/NIR	[24,91,92,93]
Cs_2_SnI_6_	Defect-variant of ABX_3_ perovskite structure	Phosphine-free hot injection	Stable films; direct bandgap (1.48 eV); high absorption coefficient; tunable morphology; tested in solar cells and FETs	[25,94]
MA_3_Bi_2_X_9_ (X = Cl, Br, I)	Distorted-layered structure	Collaborative solvent LARP (Co-LARP)	PLQY = 15% (X = Br); tunable PL peaks from 360 to 540 nm; good ethanolic stability; minor re-absorption effect	[95]
Cs_3_Bi_2_X_9_ (X = Br, Cl, I)	Vacancy-ordered ABX_3_ perovskite structure	Hot injection [96] or room T synthesis [97]	Widely-tunable absorbance; air stable	[96,97]
Cs_3_Sb_2_X_9_ (X = Cl, I)	Trigonal and orthorhombic phases	Ionic metathesis process (X = Cl)	Band-edge emission in the yellow-red (X = I); Sharp band-edge excitonic emission	[27,98]
Cs_2_InSbCl_6_	-	-	Direct bandgap (1.0 eV); stable	[99]
Cs_2_AgBiX_6_ (X = Cl, Br, I)	Elpasolite (X = Cl, Br)	Hot injection (X = Cl, Br), anion exchange reaction (X = I)	Stable, strong absorption throughout the visible region. Indirect bandgap	-
Cs_2_InSbCl_6_	-	-	Direct bandgap (1.0 eV); stable	[99]
Cs_2_AgIn*_x_*Bi_1−*x*_Cl_6_ (*x* = 0.75 and 0.9)	Fm3m cubic space group	Anti-solvent recrystallization	Direct bandgap; PLQY = 36.6%; bright dual color (violet and orange) emission	[100]

**Table 2:** Comparison the stability properties of PNCs composites. Water stability refers to samples direct placed into water. Dramatic changes imply decreasing of normalized PL to 50% of its initial value. For UV resistance, the figures in brackets are a power unit of UV radiation. “-” refers to properties not investigated in the work or measured in non-comparable conditions with respect to the others. “*” means that measurements were performed under accelerating aging conditions (high humidity level and/or temperature). “**” means that investigation was performed under sonication.

Composition	Method	PLQY of Composite, %	UV Resistance, h		Thermal Stability, °C		Water Resistance, h		Stability under Ambient Condition, Days	Ref.
			Dramatic Changes	Full Degradation	Dramatic Changes	Full Degradation	Dramatic Changes	Full Degradation		
CsPbBr_3_	Mesoporous particles (SiO_2_)	46.2	>120	>120	95	>125	>120	>120	>30	[138]
CsPbBr_3_	Mesoporous particles (SiO_2_)	-	>96 (6 W)	>96 (6 W)	>100	>100	-	-	-	[133]
Mn-doped-CsPbBr_0.5_I_2.5_	Mesoporous particles (SiO_2_)	-	-	-	-	-	-	>240	>10	[139]
Mn-doped-CsPbBr_0.5_I_2.5_	Mesoporous particles (Al_2_O_3_)	-	-	-	-	-	-	72	5	[139]
CsPbBr_3_	polyhedral-oligomeric-silsesquioxane-(POSS)	61	-	-	-	-	-	>1680	-	[185]
CsPb(Br/I)_3_		45	-	-	-	-	-	>1680	-	[185]
CsPbI_3_	Glass encapsulation (B_2_O_3_-SiO_2_-ZnO)	4.2	-	-	-	-	-	-	>8.3 *	[144]
CsPbBr_3_	Glass encapsulation (B_2_O_3_-SiO_2_-ZnO)	42.5	-	-	-	-	-	-	>60-	[145]
CsPbBr_1.5_I_1.5_	Glass encapsulation (B_2_O_3_-SiO_2_-ZnO)	15.5	-	-	-	-	-	-	>60	[145]
CsPbI_3_	Glass encapsulation (B_2_O_3_-SiO_2_-ZnO)	17.6	-	-	-	-	-	-	>60	[145]
CsPb_2_Br_5_	Glass encapsulation (ZnO-SiO_2_-B_2_O_3_)	30.6	-	-	60	>160	-	-	-	[146]
CsPbBr_1.2_I_1.8_	Glass encapsulation (P_2_O_5_-SiO_2_-ZnO)	-	>500	>500	89	180	>960	>960	>20.8	[148]
CsPbBr_3_	Glass encapsulation (P_2_O_2_-SiO_2_-ZnO)-	42	>144	>144	>125	>125	24	-	>30	[149]
CsPbBr_3_	Glass encapsulation (TeO_2_-Al_2_O_3_-H_3_BO_3_-ZnO-Na_2_CO_3_)	72	>20 (20 W)	>20 (20 W)	80	180	>1080	>1080	>45	[151]
Mn-doped-CsPbBr_1.0_I_2.0_	Glass encapsulation (B_2_O_3_-SiO_2_-ZnO)	-	-	-	81	>107	-	-	>70	[147]
CsPb_0.64_Sn_0.36_Br_3_	Glass encapsulation (B_2_O_3_-SiO_2_-ZnO)	43	-	-	140	>220	-	-	>100 *	[136]
CsPbBr_3_	Porous glass encapsulation (SiO_2_-B_2_O_3_-Na_2_O-CaO)	28	>12 (65 mW)	>12 (65 mW)	77	190	>0.17	-	-	[134]
CsPb(Cl_0.5_Br_0.5_)_3_	Porous glass encapsulation (SiO_2_-B_2_O_3_-Na_2_O-CaO)	2.6	8 (65 mW)	>12 (65 mW)	158	280	-	-	-	[134]
CsPb(Br_0.4_I_0.6_)_3_	Porous glass encapsulation (SiO_2_-B_2_O_3_-Na_2_O-CaO)	8.9	6 (65 mW)	>6 (65 mW)	54	140	-	-	-	[134]
CsPbX_3_ (X = Cl/Br, Br, I)	Porous glass encapsulation (opal matrix)	-	-	-	-	-	-	-	>30	[184]
CsPb(Cl_0.5_Br_0.5_)_3_	Polymer matrix (SBS)	10.8	-	-	-	-	0.12	1	-	[164]
CsPb(Br_0.8_I_0.2_)_3_	Polymer matrix (SBS)	23	-	-	-	-	0.17	1	-	[164]
CsPb(Br_0.6_I_0.4_)_3_	Polymer matrix (SBS)	14.6	-	-	-	-	0.1	0.5	-	[164]
CsPb(Br_0.4_I_0.6_)_3_	Polymer matrix (SBS)	12.2	-	-	-	-	0.12	0.67	-	[164]
(CsPb(Cl/Br)_3_)	Polymer matrix (ethylcellulose)	-	-	-	-	-	-	-	6	[158]
MAPbBr_3_	Polymer matrix (MMA:PMMA)	-	12 (9 W)	>24 (9 W)	-	-	-	-	-	[162]
CsPbBr_3_	Polymer matrix (PLMA EGDA)	-	14 (0.1 W/cm^2^)	>14 (0.1 W/cm^2^)	-	-	-	-	-	[163]
CsPb(Br_0.2_I_0.8_)_3_	Polymer matrix (PLMA EGDA)	-	>14 (0.1 W/cm^2^)	>14 (0.1 W/cm^2^)	-	-	-	-	>150	[163]
MAPbBr_3_	Polymer matrix (PS)	34	-	-	70	>100	-	>1440	>150	[152]
MAPbBr_3_	Polymer matrix (PC)	31	-	-	60	>180	-	>1440	>150	[152]
MAPbBr_3_	Polymer matrix (ABS)	48	-	-	75	>100	-	>1440	>150	[152]
MAPbBr_3_	Polymer matrix (CA)	47	-	-	-	-	-	>48	>150	[152]
MAPbBr_3_	Polymer matrix (PVC)	16	-	-	-	-	-	>1440	>150	[152]
MAPbBr_3_	Polymer matrix (PMMA)	-	-	-	-	-	-	0	1	[152]
MAPbBr_3_	Polymer matrix (PDMS-urea)	23.8	-	-	-	-	>216	-	-	[159]
MAPbI_3_	Polymer matrix (PDMS-urea)	-	-	-	-	-	24	>48	-	[159]
CsPbBr_3_	Polymer matrix (EVA)	40.5	>54	-	65	>75	>240	>720	>8	[161]
CsPbBr_3_	Polymer matrix (PS)	48	-	-	-	-	-	>720	-	[155]
CsPbBr_3_	Polymer matrix (PVP/silicone resin)	24	>120	-	>100	-	-	>4	>5	[165]
CsPbBr_3_	Polymer matrix (PVP/PS)	27	>10	-	-	-	-	-	-	[156]
CsPbBr_3_	Polymer matrix (SEBS)	-	-	>2.8 × 10^−4^ (50 kW/cm^2^)	-	-	-	>2928	-	[157]
CsPbBr_3_	Polymer matrix (PS)	68	288 (16 W)	>384 (16 W)	-	-	528	>6480	-	[160]
CsPbBr_3_	Polymer matrix (PMMA)	54.6	-	-	-	>80	>48	-	>30	[154]
CsPbBr_3_	Polymer matrix (PBMA)	62.2	-	-	-	-	>48	>720	>30	[154]
MAPbBr_3_	Polymer matrix (PVA)	-	>2 (2 pW)	-	-	-	-	-	-	[186]
MAPbBr_3_	Polymer matrix (PVDF)	94.6	-	>400 (6 W)	-	-	-	>400	>9 *	[187]
MAPbI_3_	Polymer matrix (PEG)	-	-	-	-	-	-	-	>12.5 *	[188]
MAPbI_3_	Polymer matrix (P123)	-	-	-	-	-	-	-	>20 *	[189]
MA_0.7_FA_0.3_PbI_3_	Polymer matrix (F127)	-	-	-	-	-	-	-	>10	[190]
CsPbMnCl_3_	Core/Shell (SiO_2_)	55.4	-	-	>100	>100	-	-	>15	[166]
CsPbX_3_	Core/Shell (SiO_2_)	60	360	>528	50	90	>24	-	-	[140]
CsPbCl_3_	Core/Shell (SiO_2_)	11.2	-	-	-	-	-	-	>30	[172]
CsPbBr_3_	Core/Shell (SiO_2_)	84	-	-	-	-	-	-	>30	[172]
CsPbI_3_	Core/Shell (SiO_2_)	45	-	-	-	-	-	-	>60	[172]
CsPb(Cl_0.5_/Br_0.5_)_3_	Core/Shell (SiO_2_)	-	-	-	-	-	2	>240	-	[172]
CsPbBr_3_	Core/Shell (SiO_2_)	-	-	-	-	-	2	>240	-	[172]
CsPb(Br_0.3_/I_0.7_)_3_	Core/Shell (SiO_2_)	-	-	-	-	-	1.3	>240	-	[172]
CsPbBr_3_	Core/Shell (SiO_2_)	90	-	-	-	-	>0.7 **	-	>28 *	[167]
CsPbBr_3_	Core/Shell (LP/SiO_2_)	90.5	>168 (8 W)	-	60	120	-	>0.6	>30	[173]
CsPbBr_3_	Core/Shell (TiO_2_)	-	>24	-	-	-	>2160	-	-	[174]
CsPbBr_3_	Core/Shell (AlO_x_)	75.3	-	-	-	-	-	>168	-	[168]
CsPbBr_3_	Core/Shell (CsPb_2_Br_5_)	-	-	-	-	-	>72	>120	-	[177]
CsPbI_3_	Core/Shell (Cs_4_PbI_6_)	-	-	-	-	-	-	-	>7	[178]
CsPbBr_3_	Core/Shell (amorphous CsPbBr_x_)	84	-	-	-	-	-	-	-	[179]
CsPbBr_3_	Core/Shell (Rb_4_PbBr_6_)	85	>10 (175 mW/cm^2^)	-	-	-	-	-	-	[176]
Mn^2+^-doped CsPbCl_3_	Core/Shell (CsPbCl_3_)	40	-	-	>110	-	-	-	-	[180]
MAPbBr_3_	Core/Shell ((C_8_H_17_NH_3_)_2_PbBr_4_)	92	-	-	-	-	-	-	>60	[181]
CsPbBr_3_	Core/Shell (PMA)	53	>12	-	-	-	-	-	-	[191]
MAPbBr_3_	Core/Shell (PMMA)	88	>7	-	-	-	-	>18	>365	[183]
MAPbBr_3_	Core/Shell (β-cyclodextrin)	89.7	>144	-	-	-	>240	-	-	[182]
MAPbI_3_	Core/Shell (PS-b-P2VP))	55	-	-	-	-	312	1800	>220	[170]

Abbreviations: PVDF—Poly(vinylidene floride); PEG—Poly(ethylene glycol); P123—Poly(ethylene glycol)-poly(propylene glycol)-poly(ethylene glycol) (PEG-PPG-PEG); F127—Poly(ethylene oxide)-poly(propylene oxide)-poly(ethylene oxide) (PEO-PPO-PEO).
